# Ambient Air Pollution and Chronic kidney disease risk in Deltan communities: A Policy Brief, 2023

**DOI:** 10.12688/f1000research.145904.1

**Published:** 2024-04-15

**Authors:** Ogochukwu Okoye, Elaine Carnegie, Luca Mora

**Affiliations:** 1 School of Health and Social Care, Edinburgh Napier University, Edinburgh, Scotland, UK; 2 Department of Medicine, Delta State University, Abraka, Delta, Nigeria; 3 Business School, Edinburgh Napier University, Edinburgh, Scotland, UK

**Keywords:** air pollution, chronic kidney disease, petrochemical industry, particulate matter, environmental health

## Abstract

Chronic kidney disease (CKD) is a persistent, devastating, yet neglected, non-communicable disease in developing and emerging countries. National, regional, and international agencies’ communications and reports on non-communicable diseases intentionally or non-intentionally do not feature CKD. The traditional risk factors for CKD, such as hypertension and diabetes, which have received relatively ample attention, do not sufficiently explain the high burden of CKD in these countries.

Ambient air pollution is an emerging significant environmental risk factor for CKD; however, epidemiological data and evidence are lacking for susceptible populations in developing countries. The Niger Delta region of Nigeria is a petrochemical hub known for environmental degradation, including air pollution, and thus, serves as a good case study for investigating the association between air pollution and CKD. This brief is based on the results of a mixed-methods study conducted in four communities situated near an oil and gas refinery in Warri, Nigeria.

Air pollutant concentrations measured in partnership with citizen scientists showed that all except one air pollutant (ozone) exceeded the WHO acceptable limits in all four communities.

The overall prevalence of CKD was high (12.3%) but even higher (18%) in a socially deprived semi-urban community closest to the oil refinery. Hypertension, diabetes, other behavioral risk factors, and exposures associated with CKD were prevalent among the inhabitants of the four communities. However, public environmental health information and education are lacking.

A multifaceted approach is required to mitigate air pollution and the associated health risks in the state. Public inclusion is strongly recommended for the planning and implementation of future interventions. Kidney disease prevention and treatment should be emphasized in health policies and insurance schemes.

## Introduction

Chronic kidney disease (CKD) is a persistent, devastating, yet neglected non-communicable disease (NCD) especially in developing and emerging countries ( Vanholder et al., 2021). Chronic kidney disease is responsible for 3.4 million deaths worldwide and ranks 10th among the risk factors for global deaths and DALYs ( Global Burden of Disease Collaboration, 2019). However, national, regional, and international agency communications and reports on non-communicable diseases intentionally or non-intentionally do not feature CKD. Traditional risk factors for CKD, such as hypertension and diabetes, which receive relatively ample attention, do not sufficiently explain the high burden of CKD in these countries ( Garcia-Garcia et al., 2015; Stanifer et al., 2016).

The disproportionately higher prevalence and severity of CKD in the young population of low- or middle-income countries (LMIC), despite preventive interventions, is a matter of global concern. Since the traditional risk factors for CKD, such as hypertension and diabetes mellitus, do not sufficiently explain the CKD epidemiology in LMIC, attention is shifting towards other environmental exposures, such as air pollution, which is the leading environmental risk factor for NCD ( World Health Organization, 2018). Systematic reviews and meta-analyses have shown that air pollution increases the risk of kidney dysfunction by 4–70%, and persons residing or working near point sources of air pollution are at an increased risk ( Okoye et al., 2022; Wu et al., 2020; Ye et al., 2021). However, these reviews were based on methodologically heterogeneous studies. In contrast, there is a proliferation of epidemiological and toxicological evidence of air pollution-associated respiratory and cardiovascular diseases. Evidence for air pollution associated with CKD is almost non-existent in Nigeria and Sub-Saharan Africa, and environmental epidemiological researchers from the Niger Delta region have stressed the general paucity of scientific evidence, advocating for research support to examine and assess the health risks associated with petroleum-related exposure ( Ordinioha & Brisibe, 2013; Orisakwe, 2021).

Coincidentally, similar to CKD, air pollution and its negative health consequences are worse in disadvantaged countries, placing an additional burden on already-strained health systems. Few reliable epidemiological studies on air pollution and kidney disease have been conducted among susceptible people living in the Niger Delta, Nigeria’s greatest petroleum hub, with CKD prevalence exceeding 10% ( Chukwuonye et al., 2018). The irreversible and progressive nature of CKD, the high prevalence and incidence rates, adverse outcomes, enormous costs of treatment, and the strain on individual and collective health costs should prompt all stakeholders to take action. The persistence of a combination of CKD and ambient air pollution (two top-ten risk factors for global deaths) despite existing environmental health regulations is concerning and deserves attention.

This document provides epidemiological evidence of air pollution associated CKD in susceptible communities in Niger Delta, Nigeria, the implications for policy and recommendations for action. The Ethical Review Committee of the Hospital Management Board, Warri, Delta State, Nigeria (CHW/ECC VOL 1/226) and the School of Health and Social Care Research Integrity Committee, Edinburgh Napier University (2782647) approved the study.

## Policy outcomes and implications

Despite the high burden of CKD in Nigeria, there is currently no renal care policy, plan or programme. Although the updated National Health Policy published in 2016 ( Federal Ministry of Health, 2016) explicitly states that all tiers of government and private sectors should commit to attaining health and good quality of life for all citizens, CKD was surprisingly not identified as one of the major NCDs requiring attention. The implication of this grave omission is that while resources are channelled towards the prevention and control of NCDs such as hypertension, cardiovascular disease, stroke and asthma, the prevalence of CKD remains high and outcomes, abysmal.

The nephrology research community are well place to generate the needed evidence that may persuade policy makers to action. This brief therefore provides epidemiological evidence of high CKD burden in susceptible communities in the Niger Delta, Nigeria and the possible association with the greatest environment risk factor for diseases - air pollution.

### Evidence of high ambient air pollutant levels in Warri

No air monitoring data existed in the State at the time this study was conducted. Ambient air pollutants were measured in collaboration with eight citizen scientists from four communities at varying distances from the petrochemical refinery: A (3 km/semi-urban), B (3.5 km/urban), C (10 km/urban), and D (13 km/rural). The mean concentrations of PM _2.5_, PM _10_, and volatile organic compounds (VOCs) exceeded the WHO acceptable limits in all four communities, whereas CO _2_ was acceptable only in the communities farthest from the refinery ( Table 1). Ozone (O _3_) was within the acceptable limits in all communities. The mean PM _10_ concentration was highest in the two communities closest to the refinery (A and B), while PM _2.5_ was highest in the urban community closest to the refinery (B). Higher than acceptable levels of NO _2_ were recorded on certain days in all communities, whereas for most other days, it was negligible. Table 1 shows the estimated hazard quotient (HQ) of the air pollutants in the four communities. The HQ was estimated by dividing the mean concentration of the individual air pollutants by their respective WHO minimum acceptable limits. An HQ ≤1 is considered a negligible hazard, while >1 indicates exposure concentrations exceeding the reference limit, but not necessarily a statistical probability of harm occurring. The calculated HQ for PM _2.5_, PM _10_, VOC, and CO _2_ based on the WHO minimum allowable limits, were elevated in all four communities.

**Table 1.** Hazard Quotient of air pollutants exceeding WHO 2021 annual acceptable limits in the four communities.

Pollutants	WHO upper limit (annual mean)	Communities A 3 km	B 3.5 km	C 10 km	D 13 km
		Mean Conc. (HQ)			
CO2 (ppm)	<600	630.58 **(1.05)**	652.41 **(1.08)**	599.04 **(1.00)**	584.25 **(0.97)**
PM _10_ (μg/m ^3^)	15	55.54 **(3.70)**	55.43 **(3.69)**	40.68 **(2.71)**	46.13 **(3.07)**
PM _2.5_ (μg/m ^3^)	5	25.32 **(5.06)**	28.01 **(5.60)**	22.82 **(4.56)**	26.18 **(5.24)**
VOC (ppm)	0.220 *	0.32 **(1.46)**	0.28 **(1.26)**	0.30 **(1.36)**	0.31 **(1.40)**
**Total HQ**		**11.27**	**11.63**	**9.63**	**10.68**

HQ=Hazard quotient, that is mean concentration/WHO upper limit; Conc.=concentration, CO _2_=carbon dioxide, PM=particulate matter, VOC=volatile organic compounds.

* Indoor threshold limit, which is typically higher than outdoor.

### High prevalence of CKD and the risk factors

A health survey was conducted over a period of six months to assess the prevalence and risk factors of CKD among 1460 community members selected by multi-stage sampling from the four communities. Adults aged 18-64 years who had resided in their respective communities for at least 5 years were recruited.

**Figure 1.**
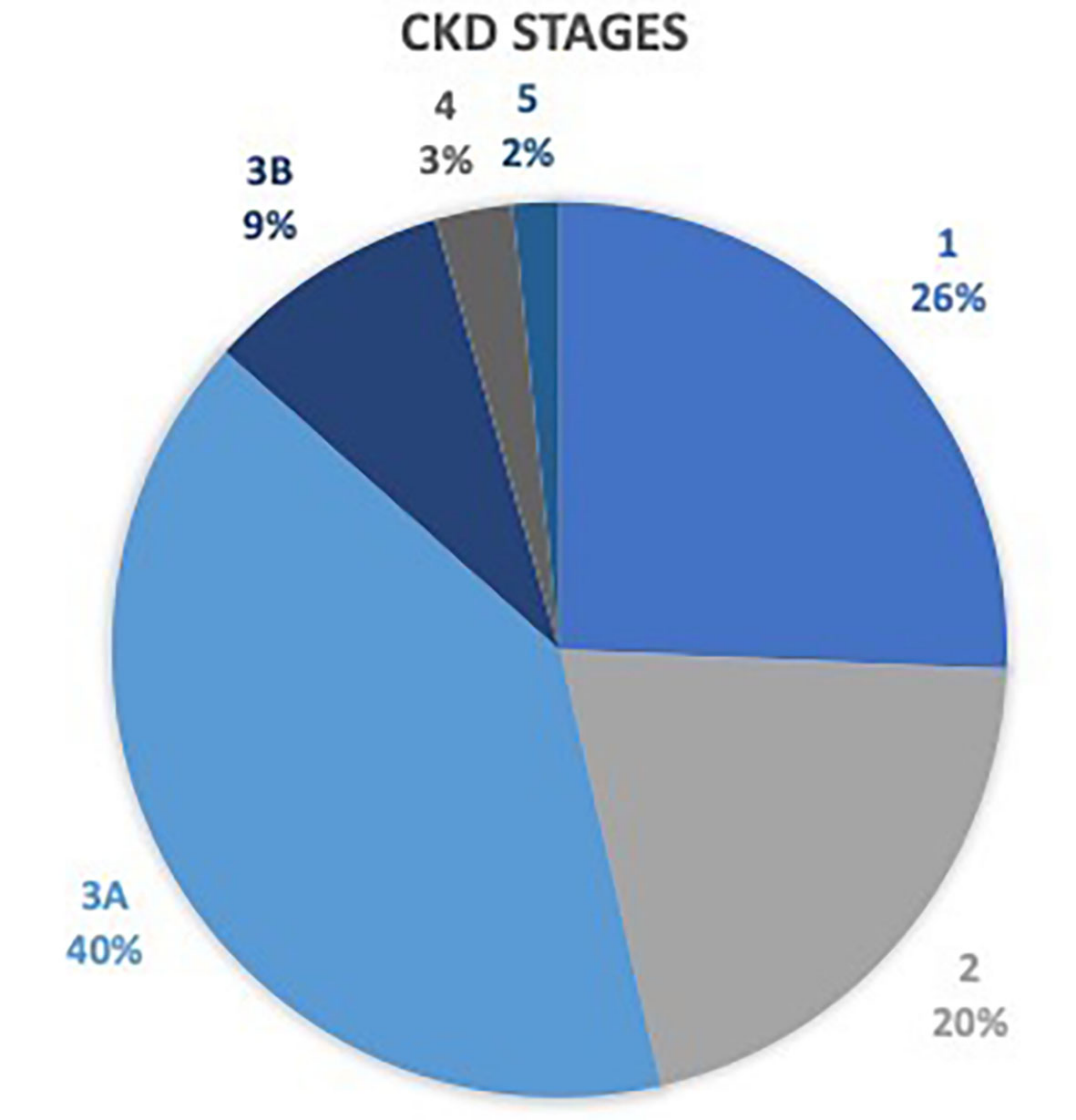
Stages of CKD among participants (N=180).

The overall prevalence of CKD, defined as dipstick proteinuria and/or an eGFR <60 ml/min was 12.3%. The prevalence was highest in community A closest to the refinery (17.9%) compared with 13.1%, 10.5%, and 8.0% in communities B, C, and D, respectively (X ^2^=18.292, p≤.001). Proteinuria alone was detected in 6.8% of all participants, while 6.6% had a reduced eGFR of <60 ml/min. Two-fifths of the participants with CKD were in stage 3A (i.e. eGFR 45-59 mls/min) which represents a mild to moderate decrease in kidney function requiring monitoring ( Figure 1).

The risk factors significantly associated with CKD were older age, low level of education, proximity to the refinery, residence in urban/semi-urban areas compared to rural areas, use of hair dyes, spending more time indoors, hypertension, and diabetes. However, after adjusting for confounding factors, the independent risk factors for CKD were *older age, low level of education, proximity to the refinery and hypertension.* The overall prevalence of hypertension, obesity, and diabetes was 33%, 28.5%, and 6.0%, respectively.

### Social determinants of CKD risk

One-third (31.5%) of the population had less than secondary-level education, and 50.5% earned less than the minimum wage. Although 86% of the population was employed, 68% were self-employed, and only 3.8% were employed by the government. Of the 68% self-employed, the majority were petty traders. Several social risk factors and toxic environmental exposures associated with CKD and NCDs were prevalent among residents of the four communities. Behavioural factors included grossly inadequate fruit (90%) and vegetable ingestion (70%), low physical activity (47.2%), use of kerosene (44%), alcohol consumption (41%), use of herbal concoctions (40.8%), and cooking with solid fuel (37.1%). Four-fifths of the population was regularly exposed to petrochemical products as part of their daily lives, 72% used household chemicals regularly, 53.2% were regularly exposed to pesticides, and 49% were exposed to toxic chemicals or dust in their jobs. Other risk factors that were relatively less prevalent included hair dye use (19%), excess salt intake (15%), use of mothballs (14.4%), use of skin lighteners (12.7%), and current smoking (3.8%).

### Low air pollution health risk literacy in Warri

**Figure 2.**
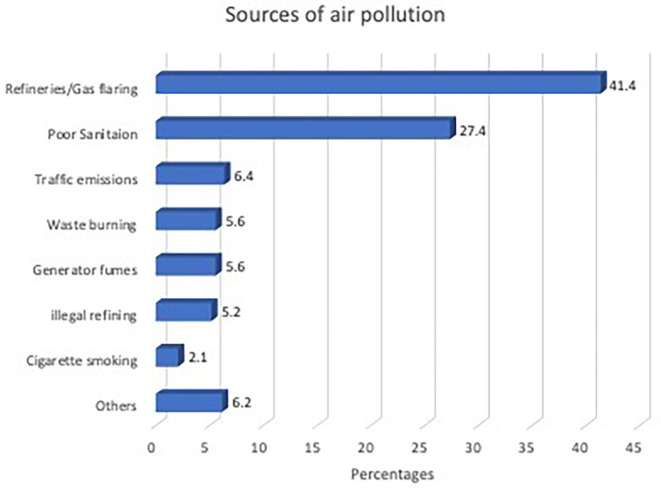
Participants’ perception of sources of air pollution (N=628). Others=Bakery, other industries, dust, overcrowding, sawmills, septic pits, swamps.

Two-fifths of the 1460 survey participants perceived that their outdoor air was polluted, and the proportion was significantly higher (65%) among those residing near the refinery. Heightened perception of air pollution was significantly more common among young people, those who lived near refineries and urban areas, those who spent more time outdoors, and those who cooked with propane gas. Refinery activities were cited as the most popular source of air pollution. A higher proportion of those residing near the refinery attributed air pollution to the refinery/gas plant: 40.6% and 18.0% for communities A and B, respectively, compared to 7.2% and 6.1% for the furthest away communities C and D, respectively. Other perceived sources of air pollution include poor environmental sanitation, traffic emissions, generator fumes, open waste burning, and illegal oil refining ( Figure 2).

**Figure 3.**
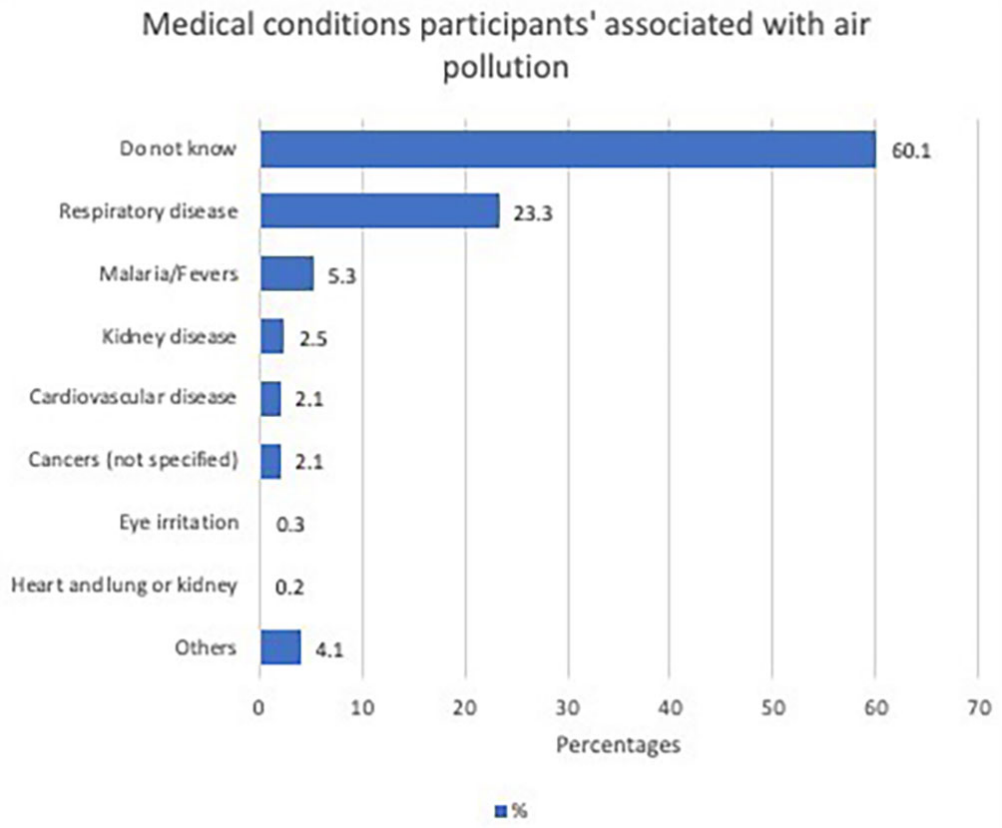
Medical conditions the participants associated with air pollution (N=1460).

Most participants (70.1%) perceived that air pollution is associated with health risks, 13.4% responded negatively, and 16.4% did not know. The majority of study participants (60.1%) were unaware of any medical conditions associated with air pollution. Figure 3 shows the participants’ responses when asked about the specific health conditions associated with air pollution.

Only 12.3% of the participants agreed that the ambient air environment was well controlled and up to 60% placed responsibility solely on the government. Among those who agreed that they had a role to play, the responses included maintaining environmental sanitation (53%), complaining to the government and advocacy (32%), and using personal protective measures (3.7%).

## Implications for policy

There is currently no renal care policy in Nigeria or Delta State, and the most recent National Health Policy does not capture CKD among NCDs. This critical omission needs to be urgently addressed as the enormous burden of CKD is not debatable. Furthermore, CKD is often associated with mortality in patients with both NCDs and chronic infections.

In the broadest policy terms, increasing efforts towards environmental risk protection, including environmental risk communication and transparency, and reducing poverty and investment in public health services, would improve population health and reduce inequalities in general, but more so for susceptible persons. Specifically, poverty and ignorance of health-promoting information increase the burden of CKD via mechanisms related to access to health care, unhealthy behaviors, biological factors (e.g., low birth weight, inadequate nutrition), and environmental factors (e.g., exposure to pollutants, communicable diseases, lack of clean water, and sanitation). Therefore, multisector integration, interdisciplinarity, and public inclusion in shaping policies and planning health interventions are needed to ensure effectiveness and reduce inequalities.

This study reveals that communities in Warri are simultaneously exposed to household, community, and global environmental risks, depicting a *Triple Risk Overlap* ( Smith & Ezzati, 2005). Due to the high prevalence of CKD risk factors and low awareness of CKD and NCD status among the study participants, it appears that health literacy is low, and the public is yet to embrace routine health screening. The implication is that current public health education interventions are not persuasive enough or effective. Out-of-pocket payments are an additional hindrance to positive health-seeking behaviors.

## Actionable recommendations

• A National Renal Care Policy is needed and should be integrated with the existing National Health Care Policy.
• Kidney health prevention and treatment should be covered by National and State health insurance schemes. Re-introducing the Delta State haemodialysis subsidy should be considered to address the suffering of people already living with kidney failure.
• All primary health centers (public and private) should be equipped to carry out basic kidney screening tests, including blood pressure monitors, urinalysis dipsticks, and portable point-of-care serum creatinine or cystatin C analyzers.
• The public requires more persuasion to adopt healthy behaviors and routine annual health screenings. For instance, a medical certificate of fitness should be required before a driver’s licence or international passport renewals.
• Environmental risk factors such as air pollution should feature prominently in strategic plans for NCD prevention. The current National Health Policy does not explicitly highlight the role of mitigating environmental exposures such as air pollution in achieving sustainable health.
• Public environmental health education is needed and should be planned and executed in collaboration with all stakeholders, including relevant government agencies, public health professionals, educators, environmental scientists, sociologists, industries, non-profit organizations, and community leaders/members. Training is required for trainers who are environmental health champions that will sustain the campaign for clean air at the community level.
• Nigeria lacks continuous air monitoring data and has met only one out of the nine Clean Air Targets ( United Nations Environment Programme, 2021).
  There is an urgent need to invest in air monitoring services and data, cleaner technologies (e.g., electric transportation, solar, and wind power), and environmental risk communication through various media channels.
• Preserve forests and green spaces. Encourage and support citizens to maintain green spaces around their homes. The Delta State Ministry of Environment has initiated a number of tree-planting campaigns, which are commendable and should be sustained.
• There is a need for socially empowering policies to improve the indices of susceptible populations that have suffered long-term environmental exposure. Job creation and skill acquisition training is required in these areas.
• All tiers of government, regulatory bodies, and urban planners should commit to improving air quality by enforcing stringent air pollution standards, regulations, and legislation. Environmental impact assessments should be conducted in accordance with ethical standards.
• There is a need to support the research community through grants to generate robust, credible, and reliable evidence that will inform effective health and social interventions. Although the National Health Policy stated the importance of “strengthening the evidence”, this can only be achieved by partnering with the experts and providing the resources to support credible research in areas of need.

### Limitations of the study

Air monitoring was conducted for a period of 4 weeks only, due to the high financial implications and tenure of the research. Urine protein was tested using dipsticks rather albumin: creatinine ratio which is more reliable, due to high cost of the test. However, dipsticks test is highly specific though less sensitive in detecting low levels of proteinuria. Lastly, the diagnosis of CKD was based on a spot- assessment due to the participants’ unwillingness to repeat their test and possibly confirm a *new disease.* The lack of a repeat test may have led to an over- or under-diagnosis of CKD.

## Conclusion

The main purpose of this briefing is to draw attention to the seriousness of chronic kidney disease, the possible causative role of environmental exposures such as air pollution, and to provide information that may support decision makers in developing and implementing policies and strategies to address the problem.

The evidence presented strongly suggests that long-term exposure to ambient air pollution increases the risk of chronic kidney disease and hypertension, which is consistent with previously published evidence. In addition, there is evidence of the socioeconomic and indirect health impacts of air pollution in an already vulnerable population with poor environmental health literacy. Environmental injustices such as disparities in access to clean air and environmental health information are significant threats to sustainable health and require urgent attention. Therefore, the co-benefits of effective air pollution mitigation surpass environmental sustainability to include improvements in health, social well-being, and reduction in health inequalities.

Addressing air pollution-associated CKD requires a multifaceted approach involving decision makers, health care professionals, the academic community, industries, and the general public. By incorporating the issue of air pollution-associated health risks into policymaking, practice, health professionals, and public education, it is possible to reduce the burden of CKD and other NCDs and improve public health outcomes.

## Data Availability

Edinburgh Napier University: Ambient Air Pollution near Petrochemical Industries and Chronic Kidney Disease Risk: Integrating Citizen Science within an Exploratory Mixed Methods Study (dataset) https://doi.org/10.17869/enu.2024.3559366. This project contains the following underlying data:
• AIR MONITORING DATA.xlsx • Codebook - air pollution - 17-03-2022.docx • GFR.xlsx Data are available under the terms of the Creative Commons Attribution 4.0 International license (CC-BY 4.0).
