# Ambient Air Pollution and Chronic kidney disease risk in Deltan communities: A Policy Brief, 2023

**DOI:** 10.12688/f1000research.145904.2

**Published:** 2024-11-21

**Authors:** Ogochukwu Okoye, Elaine Carnegie, Luca Mora

**Affiliations:** 1 School of Health and Social Care, Edinburgh Napier University, Edinburgh, Scotland, UK; 2 Department of Medicine, Delta State University, Abraka, Delta, Nigeria; 3 Business School, Edinburgh Napier University, Edinburgh, Scotland, UK

**Keywords:** air pollution, chronic kidney disease, petrochemical industry, particulate matter, environmental health

## Abstract

Chronic kidney disease (CKD) is a persistent, devastating, yet neglected, non-communicable disease, particularly in developing and emerging countries. The traditional risk factors for CKD, such as hypertension and diabetes have received relatively ample attention but do not sufficiently explain the high burden of CKD. Ambient air pollution is an emerging environmental risk factor for CKD; however, epidemiological data and evidence are lacking for susceptible populations in developing countries.

The Niger Delta region of Nigeria is a petrochemical hub known for environmental degradation, including air pollution, and thus, serves as a good case study for investigating the association between air pollution and CKD. This brief is based on an exploratory mixed-methods study conducted in four communities situated near an oil and gas refinery in Warri, Nigeria, to explore perceived and actual air pollution risks and determine whether long-term exposure to ambient air pollution is associated with CKD.

Air pollutant concentrations measured in partnership with citizen scientists using portable air sensors, showed that all except one air pollutant (ozone) exceeded the WHO acceptable limits in all four communities. PM _2.5_ ranged from 22.8 to 28.0 μg/m ^3^, PM _10_, 40.6 to 55.5 μg/m ^3^, and CO _2_, 584-652 ppm. The overall prevalence of CKD was 12.3% but even higher (18%) in a socially deprived semi-urban community closest to the oil refinery. Hypertension, diabetes, other behavioral risk factors, and exposures associated with CKD were prevalent in the four communities and environmental health information was lacking.

A multifaceted approach is required to mitigate air pollution and the associated NCD risks in the region. The government needs to invest in air monitoring services, cleaner technologies, and environmental risk communication through various media channels. We strongly recommend public inclusion in planning, designing, and implementing educational interventions. Lastly, environmental risk factors such as air pollution should feature prominently in strategic plans for NCD prevention.

## Introduction

Systematic reviews and meta-analyses have shown that air pollution increases the risk of kidney dysfunction by 4–70%, and persons residing or working near point sources of air pollution are at an increased risk ( Okoye et al., 2022; Wu et al., 2020; Ye et al., 2021). However, these reviews were based on methodologically heterogeneous studies. In contrast, there is a proliferation of epidemiological and toxicological evidence of air pollution-associated respiratory and cardiovascular diseases. Evidence for air pollution associated with CKD is almost non-existent in Nigeria and Sub-Saharan Africa, and environmental epidemiological researchers from the Niger Delta region have stressed the general paucity of scientific evidence, advocating for research support to examine and assess the health risks associated with petroleum-related exposure ( Ordinioha & Brisibe, 2013; Orisakwe, 2021).

Chronic kidney disease (CKD) is a persistent, devastating, yet neglected non-communicable disease (NCD) especially in developing and emerging countries ( Vanholder et al., 2021). Chronic kidney disease is responsible for 3.4 million deaths worldwide and ranks 10th among the risk factors for global deaths and DALYs ( Global Burden of Disease Collaboration, 2019). However, national, regional, and international agency communications and reports on non-communicable diseases intentionally or unintentionally do not feature CKD. Traditional risk factors for CKD, such as hypertension and diabetes, which receive relatively ample attention, do not sufficiently explain the high burden of CKD especially in the young population of the developing and emerging countries ( Garcia-Garcia et al., 2015; Stanifer et al., 2016). Consequently, environmental exposures such as air pollution, are increasingly being recognized as significant risk factors for NCDs ( World Health Organization, 2018).

Few reliable epidemiological studies on air pollution and kidney disease have been conducted among susceptible people living in the Niger Delta, Nigeria’s greatest petroleum hub, with CKD prevalence exceeding 10% (Chukwuonye et al., 2018). The irreversible and progressive nature of CKD, the high prevalence and incidence rates, adverse outcomes, enormous costs of treatment, and the strain on individual and collective health costs should prompt all stakeholders to take action. The persistence of a combination of CKD and ambient air pollution (two top-ten risk factors for global deaths) despite existing environmental health regulations is concerning and deserves attention.

This document is based on an exploratory mixed-methods study with embedded citizen science inquiry, conducted in four communities situated near an oil and gas refinery in Warri, Nigeria, to explore perceived and actual air pollution risk and determine whether long-term exposure to AAP increases NCD risk. Details of the initial qualitative study (a focus group) have been published ( Okoye et al., 2023), so we focus on findings from the quantitative study and citizen science inquiry. We provide epidemiological evidence of air pollution associated CKD in susceptible communities, the implications for policy, and recommendations for action. The Ethical Review Committee of the Hospital Management Board, Warri, Delta State, Nigeria (CHW/ECC VOL 1/226) and the School of Health and Social Care Research Integrity Committee, Edinburgh Napier University (2782647) approved the study.

## Policy outcomes and implications

Despite the high burden of CKD in Nigeria, there is currently no renal care policy, plan or programme. Although the updated National Health Policy published in 2016 ( Federal Ministry of Health, 2016) explicitly states that all tiers of government and private sectors should commit to attaining health and good quality of life for all citizens, CKD was surprisingly not identified as one of the major NCDs requiring attention. This lack of recognition of CKD and the environmental risk factors important in the disease epidemiology, may explain why CKD and related NCDs are persistent.

The implication of this grave omission is far reaching. While resources are channeled towards the prevention and control of NCDs such as hypertension, cardiovascular disease, stroke and asthma, it is assumed that CKD, being a consequence of these NCDs, will be automatically addressed. So far, the evidence has shown the contrary, and this is possibly because CKD is a complex syndrome with multiple aetiologies beyond the ‘usual suspects’ and also a harbinger of hypertension. The long-term consequences of continually neglecting CKD in health policies include the persistence of hypertension and CKD with associated considerable morbidity and mortality; high health care expenditure which further impoverishes the affected members of society and their families.

The nephrology research community is well place to generate the needed evidence that may persuade policymakers to action. This brief therefore provides epidemiological evidence of high CKD burden in susceptible communities in the Niger Delta, Nigeria and the association with the greatest environment risk factor for diseases - air pollution.

### Evidence of high ambient air pollutant levels in Warri

No air monitoring data existed in the State at the time this study was conducted. Ambient air pollutants were measured using portable air sensors, in collaboration with two environmental scientists and eight citizen scientists from four communities at varying distances from the petrochemical refinery: A (3 km/semi-urban), B (3.5 km/urban), C (10 km/urban), and D (13 km/rural). The air sensors were calibrated, and the citizen scientists were trained on how to use them and record their findings. For each community, two people took repeated measurements of six air pollutants over a period of 4 weeks. The geographical coordinates of each observer’s location, temperature, and relative humidity were also recorded.

**Figure 1.**
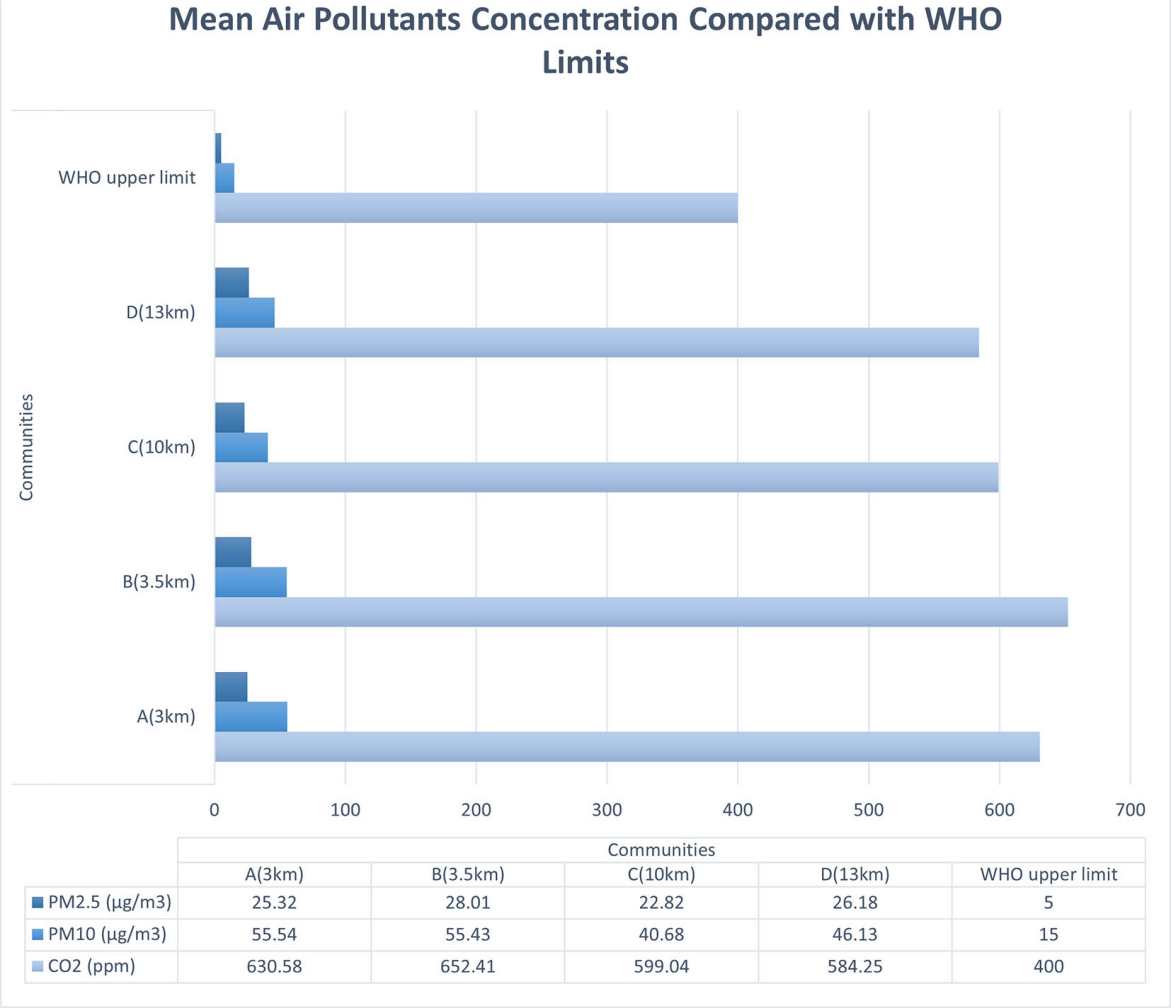
Mean air pollutant concentrations compared with acceptable limits.

The average levels of PM _2.5_, PM _10_, and volatile organic compounds (VOCs) were higher than the WHO acceptable limits in all four communities. However, CO _2_ levels were only acceptable in the communities that were the farthest away from the refinery ( Figure 1). Ozone (O _3_) was within the acceptable limits in all communities. The mean PM _10_ concentration was highest in the two communities closest to the refinery (A = 55.54 and B = 55.43 μg/m ^3^), while PM _2.5_ was highest in the urban community closest to the refinery (B = 28.01 μg/m ^3^) ( Okoye, 2024, pp. 250-254). Higher than acceptable levels of NO _2_ (>0.1-0.2 ppm) were recorded on certain days in all communities, whereas for most other days, it was negligible (0.0 or 0.1 ppm). The PM _2.5_ concentrations for three of the communities are five times higher than the WHO acceptable limit and higher than IQAir (2023) report of 23.9 μg/m ^3^ and 14.8 μg/m ^3^ for Nigeria and Warri respectively, based on estimated satellite data ( IQAir, 2023). Furthermore, the concentrations are much higher than 7.8 μg/m ^3^ achieved in Angola, the least polluted African country, which ranked 114th out of 134 countries, while Nigeria ranked the 35th most polluted.

**Figure 2.**
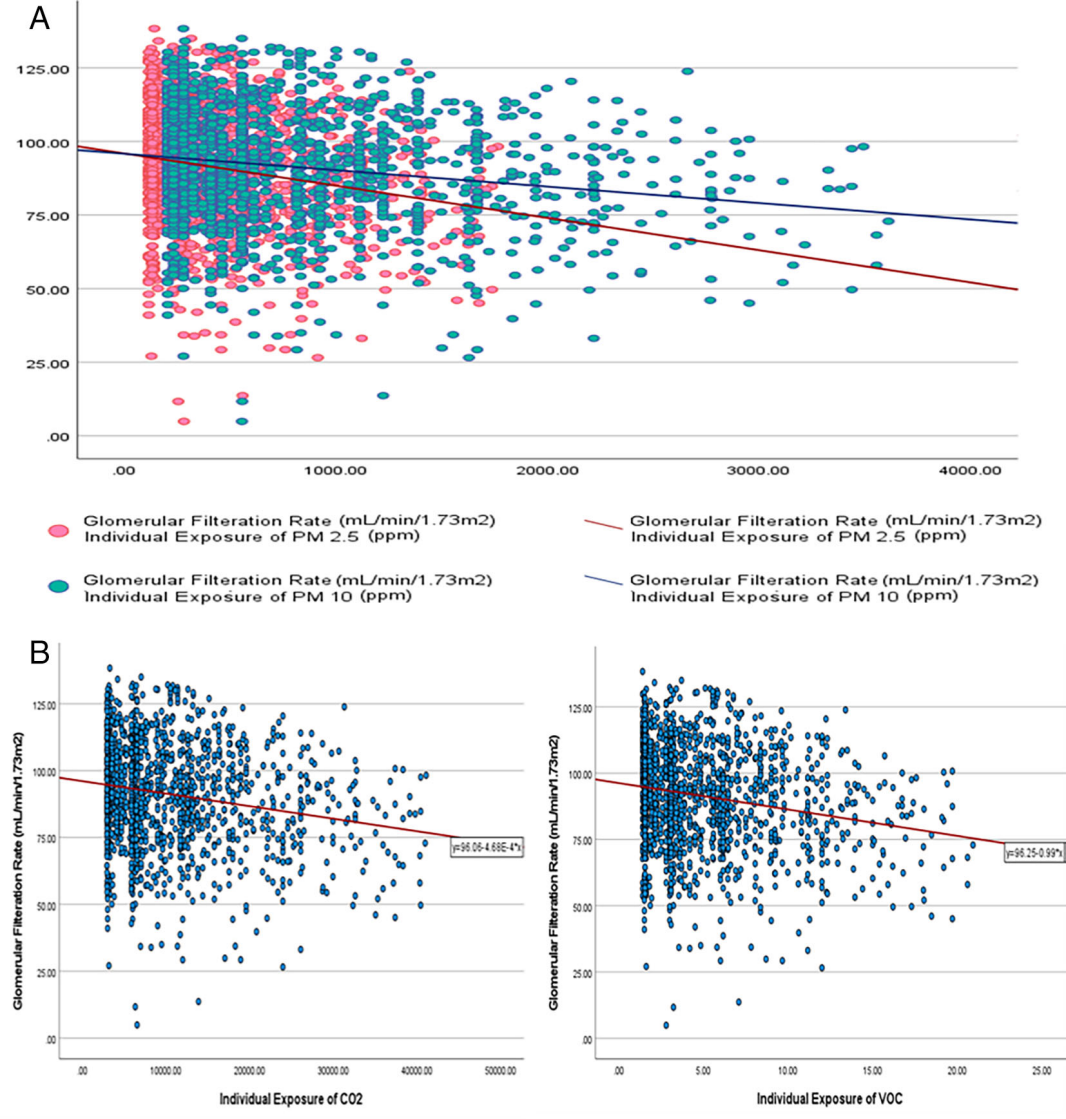
Correlation between calculated individual exposure (IE) to air pollutants and eGFR. A. Correlation between IE _PM2.5_, IE _PM10_ and eGFR. B. Correlation between IE _CO2_ and eGFR; and IE _VOC_ and eGFR.

The calculated individual exposure (IE = mean air pollutant concentration x duration of exposure) of all air pollutants was statistically significantly higher in participants who had CKD than those who did not. However, there was a weak negative correlation between estimated glomerular filtration rate (eGFR) and IEPM _2.5_, IEPM _10_, IECO _2_, and IEVOC, respectively, with rho = -0.176 to -0.190 (P<.001) ( Figure 2).

The HQ was estimated by dividing the mean concentration of the individual air pollutants by their respective WHO minimum acceptable limits. An HQ ≤1 is considered a negligible hazard, while >1 indicates exposure concentrations exceeding the reference limit, but not necessarily a statistical probability of harm occurring. The calculated HQ for PM _2.5_, PM _10_, VOC, and CO _2_ based on the WHO minimum allowable limits, were elevated in all four communities: 11.27, 11.63, 9.63, and 10.63 for communities A-D.

### High prevalence of CKD and the risk factors

A cross-sectional study was conducted over a period of six months to assess the prevalence and risk factors of CKD among 1460 community members selected by multi-stage sampling from the four communities. Adults aged 18-64 years who had resided in their respective communities for at least 5 years were recruited. The four study communities are depicted as follows: ‘A’ - nearest to refinery/semiurban, B - near/urban, C - far/urban and D - farthest/rural, to ensure participants’ privacy and anonymity. The majority of participants were female (71%) and there was no significant difference across the four communities. The mean age was 44±13 years; it was highest in far/urban and lowest in the nearest/semi-urban community, F = 11.092, df = 3, P = < .001. More than half of participants in the far/urban (66.6%) and far/rural communities were above 50 years (56%), compared with 50% respectively for the near communities.

The overall prevalence of CKD, defined as dipstick proteinuria and/or an eGFR <60 ml/min was 12.3%. The prevalence was highest in the nearest/semi-urban community (17.9%) compared with 13.1%, 10.5%, and 8.0% in the near/urban, far/urban and farthest/rural communities respectively (X ^2^=18.292, p≤.001). Proteinuria alone was detected in 6.8% of all participants, while 6.6% had a reduced eGFR of <60 ml/min. The prevalence of CKD reported across Nigeria and Sub-Saharan Africa varies greatly depending on the population studied, CKD definition and methodology; from as low as 2% to >20% ( Abd ElHafeez et al., 2018; Chukwuonye et al., 2018). However, our findings demonstrate a higher CKD prevalence in the nearest/semiurban community, compared with a pooled prevalence of 10% and 13.7% reported for Africa and Nigeria, respectively ( Abd ElHafeez et al., 2018; Raji et al., 2024).

**Figure 3.**
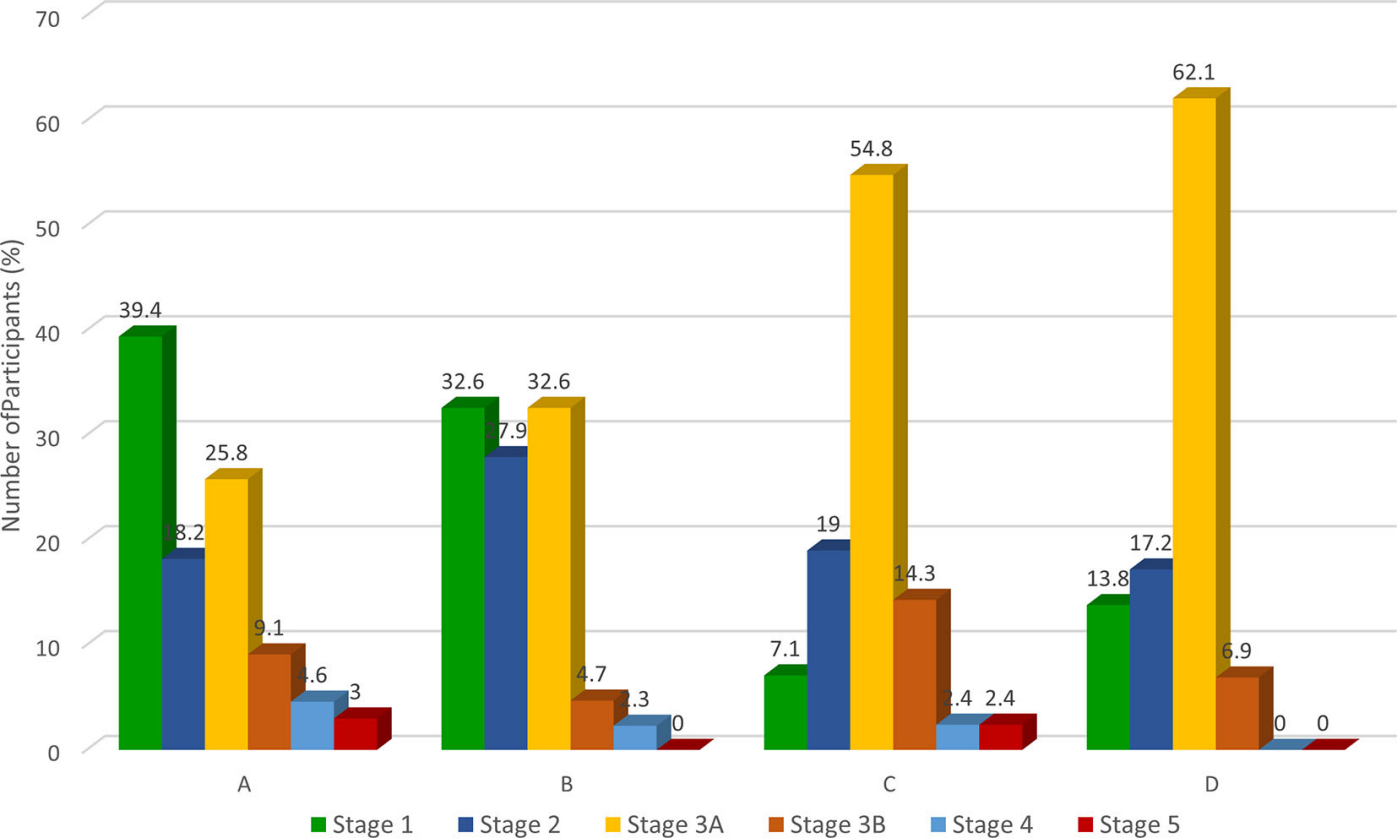
CKD staging across the four communities. *A=nearest/semi-urban| B=near/urban| C=far/urban| D=farthest/rural.

Two-fifths of the participants with CKD were in stage 3A (i.e., eGFR 45-59 mls/min) which represents a mild to moderate decrease in kidney function requiring monitoring. The nearest/semi-urban and near/urban communities had a higher proportion of participants in stage 1 and 2 CKD (proteinuria with eGFR>60 ml/min) while the far/urban and farthest/rural communities had more participants in Stage 3A ( Figure 3). Higher occurrence of proteinuria among participants closer to the refinery suggests a glomerular mechanism of kidney injury which has been previously reported ( Blum et al., 2020; Li et al., 2021; Xu et al., 2016; Yan et al., 2014). However, further experimental studies are needed to establish this. In contrast, the lower prevalence of proteinuria among the farther two communities suggests that the mechanism of kidney damage may be different. Considering that the latter two communities had an older population, aging and related comorbidities may have contributed significantly to CKD.

The risk factors significantly associated with CKD were proximity to the refinery, diabetes, hypertension, increasing age, low level of education, residence in urban/semi-urban areas compared to rural areas, use of hair dyes and spending more time outdoors. However, after adjusting for confounding factors such as gender, age, LOE, diabetes and hypertension, the independent risk factors for CKD were *proximity to the refinery* [OR=2.00 (1.43–2.81)], *increasing age* [OR=1.02(1.005–1.04)], *hypertension* [OR=1.61(1.12-2.31)], and *level of education* [OR=0.63(0.44-0.91)] ( Okoye, 2024, pp. 275). In a further logistic mixed model using R studios, which accounted for clustering effects at household level, only *increasing age* was an independent risk factor for CKD [OR=1.26 (1.09-1.45), P=.002,]; this suggests that intrahousehold homogeneity significantly accounted for the variance observed. While proximity to the refinery did not sufficiently predict CKD risk, it probably acts synergistically with other prevalent risk factors and exposures to increase the risk for CKD as explained in the multicausation theory, which is applicable to most non-communicable diseases.

The overall prevalence of hypertension, obesity, and diabetes was 33%, 28.5%, and 6.0%, respectively.

### Social determinants of CKD risk

One-third (31.5%) of the population had less than secondary-level education, and 50.5% earned less than the minimum wage. Although 86% of the population was employed, 68% were self-employed, and only 3.8% were employed by the government. Of the 68% self-employed, the majority were petty traders. Several social risk factors and toxic environmental exposures associated with CKD and NCDs were prevalent among residents of the four communities. Behavioural factors included unhealthy dietary habits (70–90%), low physical activity (47.2%), and habitual exposure to potential nephrotoxins (37–44%). Four-fifths of the population was regularly exposed to petrochemical products as part of their daily lives, 72% used household chemicals regularly, 53.2% were regularly exposed to pesticides, and 49% were exposed to toxic chemicals or dust in their jobs. Other risk factors that were relatively less prevalent included hair dye use (19%), excess salt intake (15%), use of mothballs (14.4%), use of skin lighteners (12.7%), and current smoking (3.8%).

The concentration of multiple social and environmental risk factors in the studied population may explain the high prevalence of CKD and other NCDs. These findings support the multicausation theory, drawing attention to the need for a multi-faceted approach to CKD prevention.

### Low air pollution health risk literacy in Warri

**Figure 4.**
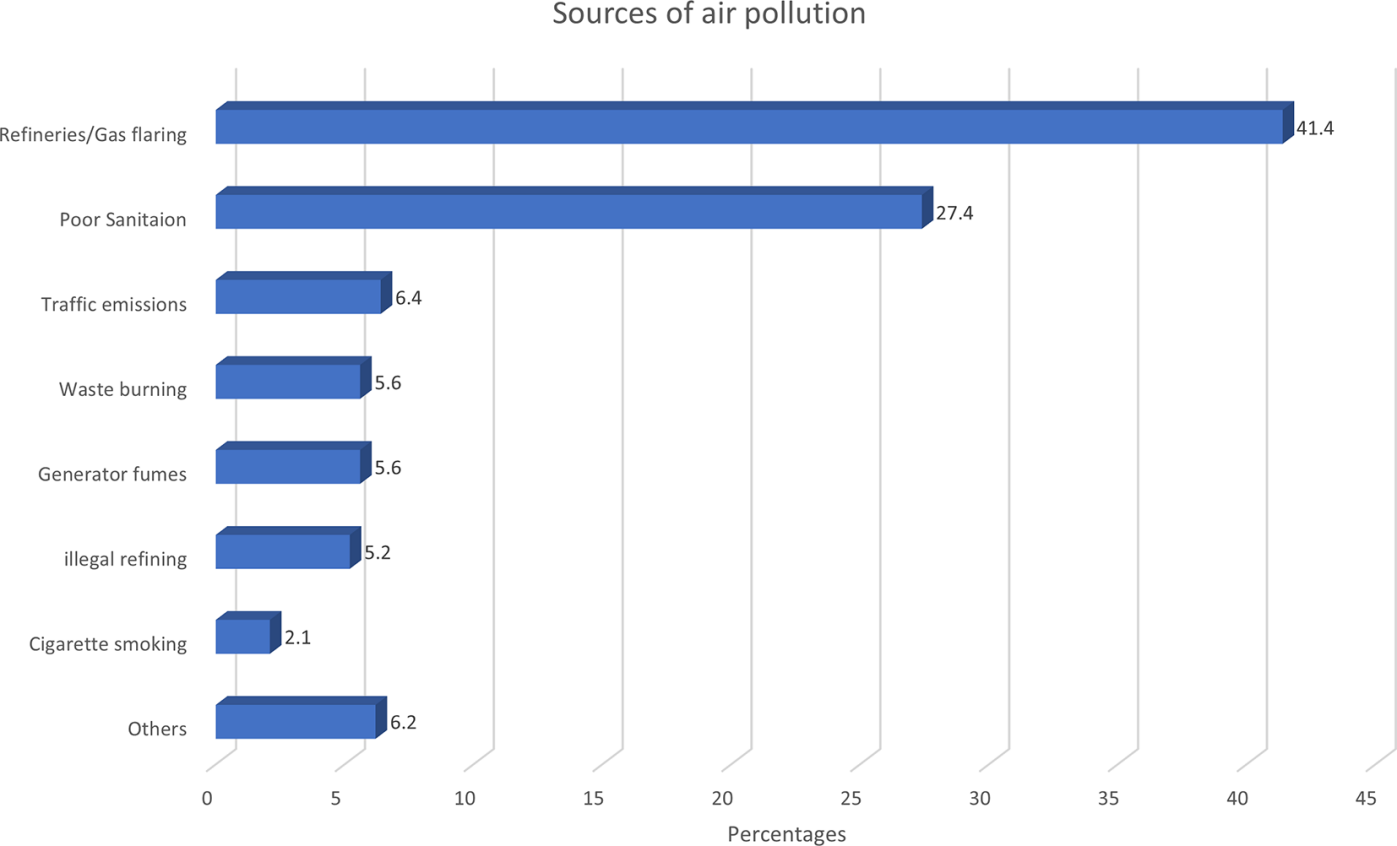
Participants’ perception of sources of air pollution (N=628). Others = Bakery, other industries, dust, overcrowding, sawmills, septic pits, swamps.

Two-fifths of the 1460 survey participants perceived that their outdoor air was polluted, and the proportion was significantly higher (65%) among those residing near the refinery. Heightened perception of air pollution was significantly more common among young people, those who lived near refineries and urban areas, those who spent more time outdoors, and those who cooked with propane gas. Refinery activities were cited as the most popular source of air pollution. A higher proportion of those residing near the refinery attributed air pollution to the refinery/gas plant: 40.6% and 18.0% for communities A and B, respectively, compared to 7.2% and 6.1% for the farther communities C and D, respectively. Other perceived sources of air pollution include poor environmental sanitation, traffic emissions, generator fumes, open waste burning, and illegal oil refining ( Figure 4).

**Figure 5.**
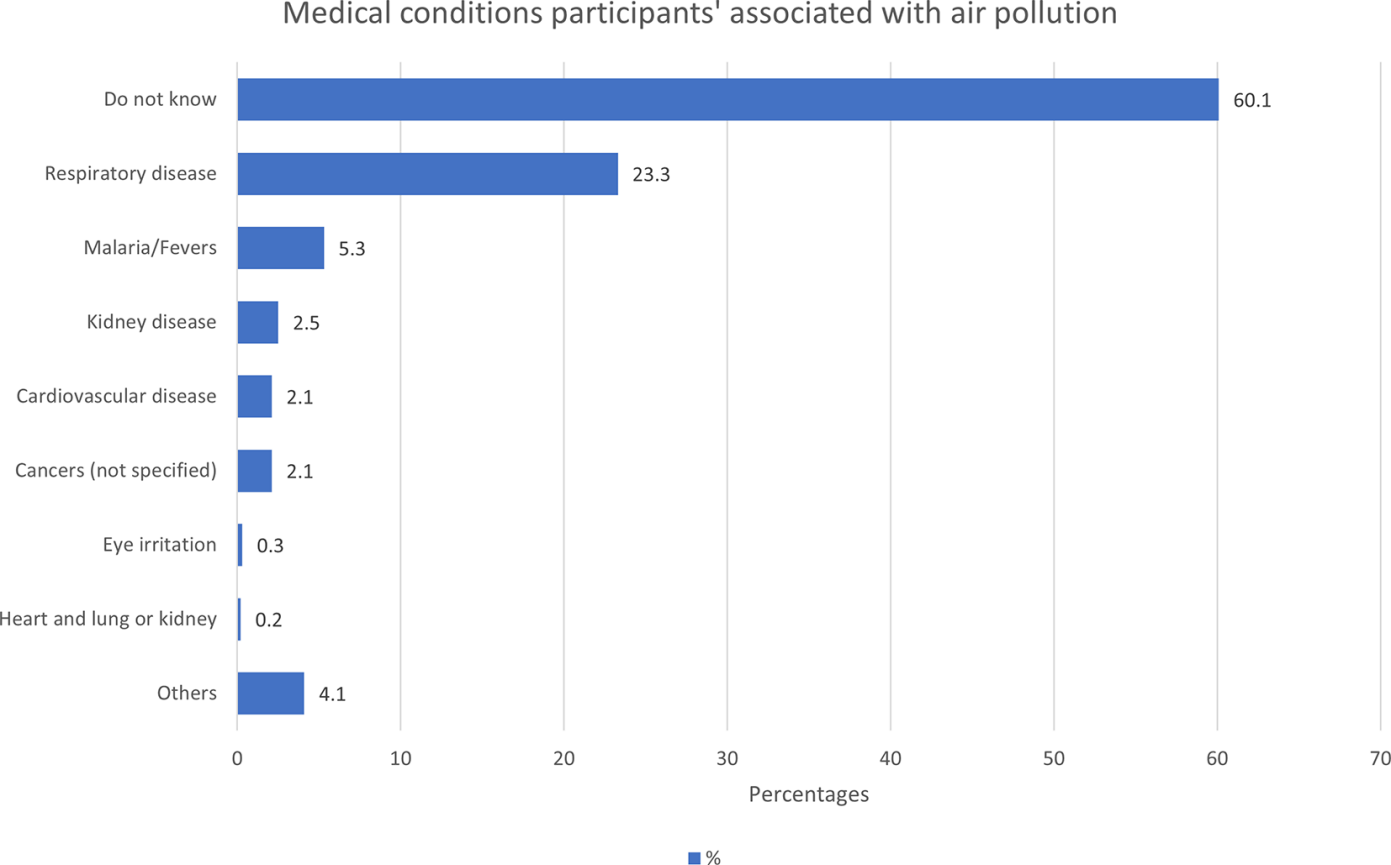
Medical conditions the participants associated with air pollution (N=1460). Respiratory disease = cough, catarrh, difficulty with breathing, chest pain, asthma, COPD, lung cancer, lung disease | Others= infections (not specified), measles, small pox, air borne disease, diarrhoea, nausea, liver disease, stomach ache, heart burn.

Most participants (70.1%) perceived that air pollution is associated with health risks, 13.4% responded negatively, and 16.4% did not know. The majority of study participants (60.1%) were unaware of any medical conditions associated with air pollution ( Figure 5). This low literacy level suggests that the necessary preventive measures, such as individual behavioural changes, are lacking, and this may contribute to the high burden of air pollution associated NCDs in the community.

Only 12.3% of the participants agreed that the ambient air environment was well controlled and up to 60% placed responsibility solely on the government. Among those who agreed that they had a role to play, the responses included maintaining environmental sanitation (53%), complaining to the government and advocacy (32%), and using personal protective measures (3.7%).

### Implications for policy

• There is currently no renal care policy in Nigeria or Delta State, and the most recent National Health Policy does not capture CKD among NCDs. This critical omission needs to be urgently addressed, as the enormous burden of CKD is not debatable. Furthermore, CKD is often the secondary cause of mortality in patients with both NCDs and chronic infections.
• Nigeria lacks continuous air monitoring data and has met only one out of the nine Clean Air Targets ( United Nations Environment Programme, 2021). Although the ministry of environment is saddled with the responsibility of monitoring and maintaining air quality, the infrastructure and equipment are lacking. However, some private organisations provide air monitoring services at a cost but more importantly, we have demonstrated that affordable portable devices are reliable and can be easily deployed to achieve the same purpose.
• In the broadest policy terms, increasing efforts towards environmental risk protection, including air monitoring, environmental risk communication, reducing poverty, and investing in public health services would improve population health and reduce inequalities, especially for susceptible persons. We have presented evidence of low environmental health literacy, low socio-economic status, and poor health indices among the communities studied, which should stimulate all stakeholders to action.
• Poverty and ignorance of health-promoting information increase the burden of CKD through mechanisms related to health care accessibility, unhealthy behaviours, biological factors (e.g., low birth weight, inadequate nutrition), and environmental factors (e.g., exposure to pollutants, communicable diseases, lack of clean water, and sanitation) ( Luyckx et al., 2017; Stanifer et al., 2016). Therefore, multisector integration, interdisciplinarity, and public inclusion in shaping policies and planning health interventions are needed to ensure effectiveness and reduce inequalities.
• Our findings reveal that communities in Warri are simultaneously exposed to household, community, and global environmental risks - a Triple Risk Overlap ( Smith & Ezzati, 2005). The high prevalence of CKD risk factors and low awareness of CKD and NCD status among the study participants suggests low health literacy and poor health-seeking behaviour, which necessitates more persuasive and inclusive public health educational interventions. Second, out-of-pocket payments are an additional hindrance to positive health-seeking behaviours and need to be addressed urgently.

Lastly, our findings are generalisable to similar vulnerable populations across the globe who reside near point emission sources. Therefore, the following recommendations may be useful for future public health interventions in these settings.

### Actionable recommendations

Table 1 below details recommendations based on our findings.

**Table 1.** Actionable recommendations, responsible stakeholders, and feasibility.

Recommendations	Responsible stakeholders	Feasibility
SHORT-TERM		
• Equip all primary health centers (public and private) to screen for CKD- blood pressure monitors, urinalysis dipsticks, and portable point-of-care serum creatinine or cystatin C analyzers.	All tiers of Govt., Non-profit organisations, Philanthropists.	PHCs already exist across the country but need upgrading.
• Public environmental health education in collaboration with all stakeholders.	Govt. agencies, public health professionals, educators, environmental scientists, sociologists, industries, non-profit organizations, and community leaders/members.	There are existing govt. public health awareness structures but need to be more inclusive from planning to intervention.
• Train the trainers who will sustain the campaign for clean air at the community level.	Govt. agencies, public health professionals, educators, environmental scientists and sociologists.	The human resources required are available but government collaboration and support is need.
• Preserved forests and maintain green spaces around residential areas.	Govt. agencies, Policy makers, environmental scientists, community members.	The Delta State Ministry of Environment has initiated a number of tree-planting campaigns, which are commendable and can be replicated across the country.
• Re-introduce the Delta State haemodialysis subsidy to address the suffering of people already living with kidney failure.	State Govt, Policy Makers.	Haemodialysis subsidy has been tried in Delta State (2013-2016) with excellent patient outcomes. Re-introduction should be strategic and transparent.
• Enforce stringent air pollution standards, regulations, and legislation. Environmental impact assessments should be conducted in accordance with ethical standards.	All tiers of Govt., regulatory bodies, and urban planners.	The standards, regulations and legislation already exist but should be enforced.
MEDIUM TERM		
• Invest in air monitoring services and data; cleaner technologies (e.g., electric transportation, solar, and wind power).	Federal and State Govt, relevant agencies (health, energy, technology), industries, environmental scientists.	Collaborate with existing private establishments and community volunteers to achieve air monitoring.
• Ensure transparent environmental risk assessment and communication.	Federal and State Govt, relevant agencies (health, energy, technology), industries, environmental scientists, educators and public health professionals.	Plan and execute risk communication strategies in collaboration with all stakeholders. Disseminate and sustain efforts through various media channels and community *Champions.*
• Persuade the public to adopt healthy behaviors and routine annual health screenings through stricter policies e.g. demand a medical certificate of fitness before driving license or international passport renewals.	Federal Govt., Policy makers.	Annual medical certificate can be obtained from accredited public and private health institutions but endorsed by only registered high-cadre health professionals.
• Support the research community through grants to generate robust evidence that will inform effective health and social interventions.	All tiers of Govt., non-profit organisations, industries, philanthropists.	The National Health Policy recognises the importance of “strengthening the evidence”. The federal govt. efforts through the Tertiary Education Trust Fund (TETFUND) is commendable but more opportunities should be created.
LONG TERM		
• A National Renal Care Policy is needed.	Federal Govt, relevant govt. agencies, policymakers, Nigerian Association of Nephrology.	The policy recommendations of the Nigerian Association of Nephrology should be adopted and integrated with the existing National Health Care Policy.
• Kidney health prevention and treatment should be covered by National and State health insurance schemes	Federal and State Govt.	The health insurance schemes already exist but need to accommodate more kidney related expenditure.
• Environmental risk factors such as air pollution should feature prominently in strategic plans for NCD prevention.	Federal Govt agencies (MOH, MOE).	The current National Health Policy does not explicitly highlight the role of mitigating environmental exposures in achieving sustainable health.
• Socially empowering policies to improve the indices of susceptible populations that have suffered long-term environmental exposure.	Federal and State Govt. and Legislators	Skill acquisition training and empowerment has been successfully implemented for certain vulnerable groups in Nigeria and can be replicated in oil and gas-situated communities.

### Limitations of the study

Air monitoring was conducted for a period of 4 weeks only due to the high financial implications and tenure of the research. Urine protein was tested using dipsticks rather than the than the albumin-creatinine ratio, which is more reliable due to the high cost of the test. However, the dipsticks test is highly specific though less sensitive in detecting low levels of proteinuria. Lastly, the diagnosis of CKD was based on a spot-assessment of urine protein excretion and eGFR and may have led to an over- or underdiagnosis of CKD. The participants’ who had abnormalities were unwilling to repeat their tests due to the fear of confirming a new disease, despite repeated attempts at inviting them through phone calls and physical visits to the community. Although this was a large sample study, the cluster size variability led to significant design effects in the prediction model.

Despite the limitations, the strength of the underlying study lies in the innovative combination of multiple research methods, interdisciplinarity and involving citizen scientists in addressing a public health problem. The extensive consideration of potential clinical, social and environmental NCD risk factors and adjusting for confounding factors strengthens the study quality.

More research is required from underserved areas to explore: this exposure-effect relationship, the mechanism of air pollution associated CKD and potential interventions to reverse it, and to develop educational interventions that would effectively improve public awareness of environmental health risks. Based on our experience with intrahousehold sample selection and the resultant design effects, we suggest that subsequent studies should consider systematic selection of households- the more, the better, and intra-household selection to ensure a constant cluster size and acceptable design effects.

## Conclusion

The main purpose of this briefing is to draw attention to the seriousness of chronic kidney disease, the possible contributory role of environmental exposures such as air pollution, and to provide information that may support decision makers in developing and implementing policies and strategies to address the problem.

Our findings show that the communities are exposed to unacceptable levels of air pollution, with a high prevalence of CKD, hypertension and other risk factors for CKD. We report that long-term exposure to ambient air pollution is associated with chronic kidney disease, which is consistent with previously published evidence. In addition, we presented evidence of the low socioeconomic indices, poor health literacy, and indirect health impacts of air pollution.

Addressing air pollution-associated CKD requires a multifaceted approach involving policymakers, health care professionals, the academic community, industries, and the general public. By incorporating air pollution-associated health risks into policymaking, clinical practice, health professionals and public education, it is possible to reduce the burden of CKD and other NCDs and improve public health outcomes.

Disparities in access to clean air and environmental health information are environmental injustice with significant threats to sustainable health and therefore require the urgent attention of policymakers. The co-benefits of effective air pollution mitigation surpass environmental sustainability to include improvements in health, social well-being, and reduction in health inequalities.

## Data Availability

Edinburgh Napier University: Ambient Air Pollution near Petrochemical Industries and Chronic Kidney Disease Risk: Integrating Citizen Science within an Exploratory Mixed Methods Study (dataset) https://doi.org/10.17869/enu.2024.3559366. This project contains the following underlying data:
• AIR MONITORING DATA.xlsx • Codebook - air pollution - 17-03-2022.docx • GFR.xlsx Data are available under the terms of the Creative Commons Attribution 4.0 International license (CC-BY 4.0).
