# Supplementary material for: Ambient Air Pollution and Chronic kidney disease risk in Deltan communities: A Policy Brief, 2023
Source: F1000Res. 2025 Jan 10;13:265. Originally published 2024 Apr 15. [Version 3] doi: 10.12688/f1000research.145904.3 (PMC11783039; doi:10.12688/f1000research.145904.3)
Supplement: Peer review history

# Reviewer report: Reviewer response for version 3

**DOI:** 10.5256/f1000research.176702.r358176

**Published:** 2025-01-16

**Authors:** Nikola M Pavlović

**Affiliations:** 1 University of Niš, Niš, Serbia

The authors have followed my instructions and comments, and I believe the article is now suitable for indexing. I have no further requests for revisions.

Does the paper provide a comprehensive overview of the policy and the context of its implementation in a way which is accessible to a general reader?

Yes

Is the discussion on the implications clearly and accurately presented and does it cite the current literature?

Yes

Are the recommendations made clear, balanced, and justified on the basis of the presented arguments?

Yes

Reviewer Expertise:

NA

I confirm that I have read this submission and believe that I have an appropriate level of expertise to confirm that it is of an acceptable scientific standard.

# Reviewer report: Reviewer response for version 2

**DOI:** 10.5256/f1000research.172808.r348567

**Published:** 2024-12-30

**Authors:** Karl Kilbo Edlund

**Affiliations:** 1 Occupational and Environmental Medicine, School of Public Health and Community Medicine, Institute of Medicine, Sahlgrenska Academy, University of Gothenburg, Göteborg, Sweden

Okoye and colleagues have conducted a much-needed study of air pollution and CKD risk in communities residing near a petrochemical plant in the Niger river delta in Nigeria. They present timely policy advice that combines previous evidence and their own findings of air pollution levels, population health, population health literacy, and the relationship, in their data, between air pollution exposure and prevalent CKD. The authors highlight the need for expanded care options for Nigerans with CKD as well as the urgent need of reduction of PM emissions from the petrochemical industry. The text is mostly well-written and the recommendations mostly rest on a solid scientific literature. I believe their implementation would benefit Nigerians much along the lines described by the authors.

 In this review, my main concerns relate to 1) the inclusion of considerable previously unpublished empirical data and analyses in this policy brief, which in this genre should rather be kept at a minimum, 2) the formulation of some of the actionable policy recommendation require further work. At the end of the review, I also include some minor comments for the authors’ consideration.

 Starting with the first concern, regarding the inclusion of primary data and analyses in this policy brief. As highlighted by the authors, there is considerable previous evidence of adverse effects on kidney health associated with air pollution, and even some evidence specifically for petrochemical industry. This means that, in the context of this policy brief (notably unlike if this were an original article or a dissertation) the empirical, quantitative evidence put forth here do not serve the purpose of testing the hypothesis that there are adverse effects on kidney health of air pollution, but rather to emphasise that the adverse health effects seen elsewhere are highly likely to extend to the Niger river delta as well. In the first scenario, the data provided here, although filling an important gap in previously available data and describing the high prevalence of undiagnosed CKD in this population, do not provide particularly strong evidence for adverse kidney effects of PM from the petrochemical industry, above and beyond what is already available. Key limitations that need to be kept in mind are that these data are cross-sectional, that participant selection and sampling procedures are not described, that PM measurements were taken only during certain times and through a less standardised process and that no validation is described etc. (These limitations are of course fully understandable given the challenges of data collection.) On the other hand, these data are strong when instead considered as a validation that the likely large impact of PM on kidney health noted in previous literature also applies to this area.

 With this in mind, I would suggest the authors restructure the section (“High prevalence of CKD and risk factors”) where the association between air pollution and CKD is presented. Firstly, it would help the reader if this rather long section were subdivided, as it currently presents the two rather different topics of CKD prevalence and then the PM-CKD association. Secondly, I believe that it would lend more strength to the authors’ recommendations, if the authors would present a little more extensively the rapidly growing literature supporting an air pollution-CKD association, with particular note to any previous studies in similar settings. Thirdly, I think the authors should reconsider the currently rather technical discussion on regression analyses and statistical methodologies, given that this is presumably of little use to a policy-maker who is not themselves trained in quantitative analysis; possibly, it suffice to say that the prevalence of CKD was higher closer to the refineries adjusting for age, sex, BMI, if this is in line with how the authors interpret their results.

 Turning to the second concern, relating to the policy recommendations. The conclusions bullet-pointed under “Implications for policy” are substantive, well-written, and supported by literature and empirical findings. However, I have concerns with multiple recommendations provided as actionable recommendations in table 1. In the following, I will for practical reasons refer to them as numbers, with recommendation 1 as the first recommendation in the table.

- For recommendation 1, the call for CKD screening is a charitable one. However, the decision on initiating screening is a difficult one, and should include a cost-benefit analysis as well as strategies to accurately identify individuals with false-positive screening results. Furthermore, this analysis needs to account for the availability of treatment options.
- Similarly, the subsidy reintroduction mentioned in recommendation 5 would need some cost-benefit analysis, or if such is available elsewhere, as a reference.
- The suggestion of a medical fitness certificate before international passport renewals in recommendation 9 might not be compatible with the UN’s Universal Human Rights (article 13, section 2). However, this is beyond my area of expertise.
- The National Renal Care Policy referred to in recommendation 11 is not mentioned or detailed in the policy brief, and for the non-Nigerian it might be difficult to know what the authors refer to here.
- Including environmental risk factors in NCD prevention plans, as suggested in recommendation 13, could be supported by an attributable fraction calculation, illustrating the magnitude of these risk factors in comparison to other relevant, traditional risk factors.

Minor comments:

- The air pollutant levels, measured during 4 weeks, are compared to the annual WHO limits. While this is presumably necessary due to lack of more extensive data, it would be recommended to mention this.
- Hazard quotients (HQ) are presented for PM _2.5_, PM _10_, VOC and CO _2_, no concentration or WHO limit is presented for VOC. Furthermore, CO _2_, while central to global warming, is not a criteria pollutant and there is little literature to support adverse health effect of direct CO _2_ exposure. It also seems, purely from the figures themselves, that HQs for the pollutants have been summarised to a combined HQ, and it is questionable whether this method can capture magnitudes of and synergies between health effects in any meaningful way.
- The in-text link is missing for a key reference in the Introduction (Chukwuonye et al., 2018). Furthermore, the “Publisher Full Text” link in the first author’s thesis redirects not to the thesis but to a conference abstract.
- The authors report using “R studios” for statistical analyses, yet it should be noted that the RStudio software is merely a front-end for R, which is the statistical programme used. If the authors wish to state their choice of statistical software in this policy brief (see the first major concern outlined above), I would recommend stating it more correctly as R.

Does the paper provide a comprehensive overview of the policy and the context of its implementation in a way which is accessible to a general reader?

Partly

Is the discussion on the implications clearly and accurately presented and does it cite the current literature?

Partly

Are the recommendations made clear, balanced, and justified on the basis of the presented arguments?

Partly

Reviewer Expertise:

Environmental epidemiology

I confirm that I have read this submission and believe that I have an appropriate level of expertise to confirm that it is of an acceptable scientific standard, however I have significant reservations, as outlined above.

# Response

**Published:** 2024-12-30

**Authors:** Ogochukwu Okoye

**Affiliations:** Medicine, Delta State University, Abraka, Delta, Nigeria

We appreciate your review and useful critique. 

 We acknowledge your main concerns and wish to respond here. However, we will revise our manuscript where needed.

 1) Regarding the inclusion of considerable empirical data and analysis:   The original manuscript submitted had minimal empirical data as expected for a policy brief. However, we included more data in response to two of the previous reviewers. We agree that the intended audience may find some aspects of the manuscript too technical and we will make some adjustments to minimise the data and complexity.

 Although the empirical data has not been published in any journal, we included a link to the Thesis underlying this study. We agree that our data serves to support the existing literature on PM and kidney disease, although these studies are mostly from the global north.  While we note the study limitations, we agree that our findings are a strong validation that air pollution associated health effects noted elsewhere also applies to the Niger Delta. 

 2) The section on "High prevalence of CKD and risk factors"  will be split to improve clarity. 

 3) Response to concerns about some 'actionable recommendations: 

 a)We agree that whole population screening may be cost-intensive and require cost-benefit analysis. However, screening of high-risk populations is currently recommended in Nigeria and globally. (KDIGO). 

 Treatment options for early CKD mostly entails addressing identified risks to slow progression of the disease. These treatment measures are widely available. 

 b)We agree that the subsidy re-introduction would need a cost-benefit-analysis, which we consider a great suggestion for further studies. 

 c) Thank you for drawing our attention to the possible Human Rights implication of requiring a medical fitness certificate before issuing international passport. We will explore the feasibility and revise this accordingly.

 d) Regarding the calculated Hazard Quotients and Total HQ, we stated that the figures do not necessarily represent  a statistical probability of harm occurring. However, we have included a further statement that 'the Total HQs do not represent synergies or magnitude of health effect'. 

 e) The concentration for VOC was not included in Figure 1, for simplicity of presentation as the values were less than 1. However, the HQs for VOC were included in the calculation of total HQ. Again, for simplicity, we have not included these details in the text. All information is available in the underlying Thesis. 

 f) The two references highlighted will be sorted. Thank you. 

 g) We will replace 'R studios' with 'R'. Thank you

# Reviewer report: Reviewer response for version 1

**DOI:** 10.5256/f1000research.159925.r279715

**Published:** 2024-09-14

**Authors:** Nicholas Osborne

**Affiliations:** 1 The University of Queensland, Saint Lucia, Queensland, Australia

**Overall comments**

 This study proposes to discover association between air pollution and CKD. Unfortunately an accurate measure of air pollution was not available. Similarly an accurate measure of the health outcome, kidney function was not available.  Diagnosis of CKD requires two measures of kidney function (usually by eGRF) at least 3 months apart. Consequently attempts to examine the relationship do not have data of sufficient quality to make the linkage.

 The paper was not conducted as a policy brief.  Large amounts of extant literature were missing from the evidence base presented.

 The paper has a limited data reference list.

**Specific comments**

 Abstract mentions CKDu in passing without mentioning its prevalence in certain LMIC settings.  The disease CKDu in Sri Lankan and Mesoamerican settings.

Does the paper provide a comprehensive overview of the policy and the context of its implementation in a way which is accessible to a general reader?

No

Is the discussion on the implications clearly and accurately presented and does it cite the current literature?

No

Are the recommendations made clear, balanced, and justified on the basis of the presented arguments?

No

Reviewer Expertise:

Environmental epidemiology

I confirm that I have read this submission and believe that I have an appropriate level of expertise to state that I do not consider it to be of an acceptable scientific standard, for reasons outlined above.

# Response

**Published:** 2024-09-30

**Authors:** Ogochukwu Okoye

**Affiliations:** Medicine, Delta State University, Abraka, Delta, Nigeria

We are grateful for your detailed and constructive critique which has helped to significantly improve the merit and quality of our manuscript. 

**Comment:** This study proposes to discover association between air pollution and CKD.  Unfortunately, an accurate measure of air pollution was not available. 

 Consequently, attempts to examine the relationship do not have data of sufficient quality to make the linkage.

**Response:** Extensive methodological and statistical details were deliberately omitted as this document was targeted at policymakers. However, we have provided additional information in the text  to describe how air pollution was measured and other details are available in the link to the underlying study.  Page 4

**Comment: **Similarly, an accurate measure of the health outcome, kidney function was not available.

 Diagnosis of CKD requires two measures of kidney function (usually by eGRF) at least 3 months apart.

**Response:** We agree with your observation. The authors initially set out to obtain at least two measurements from participants who have abnormalities but encountered challenges with participant retention. This limitation has been highlighted in more detail. 

 However, all biological samples were handled and processed by a reputable independent laboratory with documented standard operating procedures to ensure accuracy and reliability.  Page 11

**Comment: **The paper was not conducted as a policy brief. Large amounts of extant literature were missing from the evidence base presented **.**

**Response: **Thank you for your valid observation. We structured the paper according to the journal's template for a policy brief. Our intention was to provide a brief summary of the underlying study before delving into the policy implications and recommendations. We deliberately excluded excessive statistical information to enhance readability for the non-academic audience. However, we have now included more data in the text figures and supplementary files. 

**Comment: **The paper has a limited data reference list.

**Response: **The short reference list was to adhere to the limits of a policy brief. More references have been included in specific sections. Pages 3,5,6,7,14

**Comment: **Abstract mentions CKDu in passing without mentioning its prevalence in certain LMIC settings.  The disease CKDu in Sri Lankan and Mesoamerican settings.

**Response: **Thank you for your comment. There may have been a mix up. CKDu was not mentioned in the abstract.

 -

# Reviewer report: Reviewer response for version 1

**DOI:** 10.5256/f1000research.159925.r292274

**Published:** 2024-09-11

**Authors:** Nikola M Pavlović

**Affiliations:** 1 University of Niš, Niš, Serbia

**Ambient Air Pollution and Chronic kidney disease risk in Deltan communities: A Policy Brief, 2023.**

**Abstract**

- **Comments:**
  - The abstract effectively highlights the importance of the study by addressing an issue that is often overlooked: CKD in developing countries. However, it could refine its focus on the study's objectives, methods, key findings, and specific policy recommendations.
- **Suggestions:**
  - **Objective Clarification:** Include a brief statement that clearly outlines the study's objective.
  - **Methodology Mention:** Explicitly mention that a mixed-methods approach was used.
  - **Specific Findings:** Highlight specific data points, such as the exact levels of pollutants that exceed WHO limits.
  - **Policy Recommendations:** Summarize one or two key policy recommendations for a clear takeaway.

**Introduction**

- **Comments:**
  - The introduction is comprehensive, providing a strong background on CKD and air pollution, particularly in the context of developing countries. However, it somewhat repeats itself by discussing traditional CKD risk factors.
- **Suggestions:**
  - **Streamlining:** Consider consolidating the discussion on traditional CKD risk factors to avoid redundancy.
  - **Focused Transition:** Ensure a smooth transition from discussing CKD globally to the specific context of the Niger Delta.
  - **Identification of Gap:** Introduce the research gap on the link between air pollution and CKD in the Niger Delta earlier in the section.

**Policy Outcomes and Implications**

- **Comments:**
  - This section succinctly outlines the critical issue of lacking renal care policies in Nigeria. However, the link between CKD and air pollution could be articulated more forcefully to emphasize urgency.
- **Suggestions:**
  - **Direct Link:** Explicitly connect the absence of CKD in national health policy to the study’s findings on air pollution and CKD prevalence.
  - **Broader Implications:** Discuss the broader public health implications if this policy gap continues, including potential long-term consequences.

**Evidence of High Ambient Air Pollutant Levels in Warri**

- **Comments:**
  - This section is rich in data and provides a clear picture of pollution levels. The use of a table to present hazard quotients is effective, but the narrative could be enhanced for better flow and readability.
- **Suggestions:**
  - **Narrative Flow:** Integrate the table with the narrative by referencing key figures directly in the text.
  - **Benchmark Against Global Standards:** Briefly compare the findings with global or regional averages for context.
  - **Graphical Representation:** Consider using a graph to visualize the levels of pollutants across communities for greater impact.

**High Prevalence of CKD and the Risk Factors**

- **Comments:**
  - This section effectively presents prevalence data and associated risk factors. The statistical significance is clearly noted, strengthening the findings.
- **Suggestions:**
  - **Risk Factor Hierarchy:** Present the risk factors in order of their strength of association with CKD.
  - **Visual Aids:** While the current figure is useful, additional visual aids, such as bar charts or scatter plots, could further clarify the distribution of CKD stages across communities.
  - **Contextualization:** Briefly contextualize the CKD prevalence data with national or regional statistics to highlight the severity.

**Social Determinants of CKD Risk**

- **Comments:**
  - The social determinants are well-captured, providing a comprehensive picture of contributing factors. However, the section could benefit from a more concise presentation.
- **Suggestions:**
  - **Condensation:** Consider condensing the list of behavioral factors by grouping similar items, such as unhealthy dietary habits.
  - **Clearer Connections:** Draw clearer connections between these social determinants and the higher CKD prevalence, emphasizing the compounding effect of multiple risk factors.
  - **Implications:** Discuss the potential public health implications of these social determinants in more depth.

**Low Air Pollution Health Risk Literacy in Warri**

- **Comments:**
  - This section addresses an important issue but could be more impactful if it connected low health literacy directly with CKD prevalence.
- **Suggestions:**
  - **Direct Linkage:** Explicitly link low literacy levels to poor health outcomes, particularly in the context of CKD and air pollution.
  - **Awareness Campaigns:** Suggest potential public health campaigns or educational interventions to improve awareness based on the findings.

**Implications for Policy**

- **Comments:**
  - This section provides a strong call to action. The identified policy gaps are critical and well-presented.
- **Suggestions:**
  - **Structured Recommendations:** Present the policy implications in a more structured format, possibly using bullet points or numbered lists for clarity.
  - **Evidence Integration:** Integrate specific study findings more directly into the policy recommendations to strengthen the argument for immediate action.

**Actionable Recommendations**

- **Comments:**
  - The recommendations are comprehensive and relevant but could be more prioritized or categorized.
- **Suggestions:**
  - **Prioritization:** Consider categorizing the recommendations into short-term, medium-term, and long-term actions.
  - **Stakeholder Engagement:** Emphasize the role of specific stakeholders (e.g., government, NGOs, the public) in implementing these recommendations.
  - **Feasibility:** Briefly discuss the feasibility of these recommendations, particularly in the context of existing infrastructure and resources.

**Limitations of the Study**

- **Comments:**
  - The limitations are transparently discussed, which is commendable. However, there is a risk that some of these might overshadow the study's strengths.
- **Suggestions:**
  - **Strengths Emphasis:** Counterbalance the limitations with a brief discussion of the study’s strengths, particularly its novel approach and the involvement of citizen scientists.
  - **Future Directions:** Suggest specific areas for future research that could address these limitations, enhancing the study's impact.

**Conclusion**

- **Comments:**
  - The conclusion effectively summarizes the key points but could be more emphatic in calling for urgent action.
- **Suggestions:**
  - **Stronger Call to Action:** End with a more forceful call to action directed at policymakers, highlighting the urgency of addressing CKD in the Niger Delta.
  - **Summary of Key Points:** Briefly summarize the most critical findings and recommendations to leave a lasting impression.

**Overall Document Comments:**

- **Consistency:** Ensure consistency in the use of terms (e.g., CKD vs. chronic kidney disease) throughout the document.
- **Clarity:** While the document is generally clear, some sections, particularly those with dense data, could benefit from simpler language and clearer transitions.
- **Visual Aids:** Consider the strategic use of additional visual aids to break up text-heavy sections and improve reader engagement.
- **Proofreading:** A thorough proofreading is recommended to correct any minor grammatical errors and ensure a polished final submission.

I believe these suggestions will assist the authors in refining their policy brief to make it more impactful and ensure it effectively communicates the urgency of the issue to both policymakers and the broader public.

Does the paper provide a comprehensive overview of the policy and the context of its implementation in a way which is accessible to a general reader?

Yes

Is the discussion on the implications clearly and accurately presented and does it cite the current literature?

Yes

Are the recommendations made clear, balanced, and justified on the basis of the presented arguments?

Yes

Reviewer Expertise:

NA

I confirm that I have read this submission and believe that I have an appropriate level of expertise to confirm that it is of an acceptable scientific standard.

# Response

**Published:** 2024-09-30

**Authors:** Ogochukwu Okoye

**Affiliations:** Medicine, Delta State University, Abraka, Delta, Nigeria

We are grateful for your detailed and constructive critique which has helped to improve the merit and quality of our manuscript.    

 ABSTRACT

**Reviewer comment: **Objective Clarification: Include a brief statement that clearly outlines the study's objective.

**Author response: **The objective has been clarified as follows:

 “This brief is based on a mixed-methods study conducted in four communities situated near an oil and gas refinery in Warri, Nigeria to explore perceived and actual air pollution risk and determine whether long-term exposure to AAP increases NCD risk” (Page 2)

**Reviewer comment:  **Methodology Mention: Explicitly mention that a mixed-methods approach was used.    

**Author response: **Done as stated above  

**Reviewer comment: **Specific Findings: Highlight specific data points, such as the exact levels of pollutants that exceed WHO limits.    

**Author response: **Included as follows:

 “Measured PM _2.5_ ranged from 22.8 – 28.0 μg/m3, PM _10_, 40.6 - 55.5 μg/m3, and CO _2_, 584-652ppm”.    2

**Reviewer comment: **Policy Recommendations: Summarise one or two key policy recommendations for a clear takeaway.****

**Author response: **Included as follows: 

 “The government needs to urgently invest in air monitoring services and data, cleaner technologies and environmental risk communication through various media channels. We strongly recommend public inclusion in planning, designing and implementing future environmental interventions. Lastly, environmental risk factors such as air pollution should feature prominently in strategic plans for NCD prevention”. (Page 2)

**Introduction**   

**Reviewer comment: **Streamlining: Consider consolidating the discussion on traditional CKD risk factors to avoid redundancy.  

 Focused Transition: Ensure a smooth transition from discussing CKD globally to the specific context of the Niger Delta.

 Identification of Gap: Introduce the research gap on the link between air pollution and CKD in the Niger Delta earlier in the section.  

**Author response: **Thank you for your suggestions. We have reviewed this section accordingly. (Page 3)

**Reviewer comment: **Policy Outcomes and Implications

**Author response: **This section has been improved accordingly

**Reviewer comment: **Direct Link: Explicitly connect the absence of CKD in national health policy to the study’s findings on air pollution and CKD prevalence.    

**Author response: **This has been revised as follows: “This lack of recognition of CKD and the environmental risk factors important in the disease epidemiology, may explain why CKD and related NCDs are persistent.” (Page 4)

**Reviewer comment: **Broader Implications: Discuss the broader public health implications if this policy gap continues, including potential long-term consequences. ** **  

**Author response: **“The long-term consequences of continually neglecting CKD in health policies include the persistence of hypertension and CKD with associated considerable morbidity and mortality; high health care expenditure which further impoverishes the affected members of society and their families”.   (Page 4)

**Reviewer comment: **Evidence of High Ambient Air Pollutant Levels in Warri        

 Narrative Flow: Integrate the table with the narrative by referencing key figures directly in the text.    

**Author response: **This section has been improved accordingly and highlighted in the text        ( Page 4-5)

**Reviewer comment: **Benchmark Against Global Standards: Briefly compare the findings with global or regional averages for context.    

**Author response: **This section has been improved as follows: 

 “The PM2.5 concentrations for three of the communities are higher than Nigeria’s 2023 average of 23.9 μg/m3 (IQAIR) and five times higher than the WHO acceptable limit. Furthermore, these levels are much higher than 7.8μg/m3 achieved in Angola, the least polluted African country which ranked 114th out of 134 countries, while Nigeria was ranked the 35th most polluted”.( Page  4-5)

**Reviewer comment: **Graphical Representation: Consider using a graph to visualize the levels of pollutants across communities for greater impact.    

**Author response: **A bar chart showing air pollutant levels in the 4 communities have been included. (Figure 1)    

**Reviewer comment: **High Prevalence of CKD and the Risk Factors        

 Risk Factor Hierarchy: Present the risk factors in order of their strength of association with CKD.    

**Author response: **Included as follows: 

 “The independent risk factors for CKD were proximity to the refinery [OR=2.00 (1.43 – 2.81], increasing age [OR=1.02(1.005-1.04)], hypertension [OR=1.61(1.12-2.31)], and level of education [OR=0.63(0.44-0.91)]. In a further logistic mixed model, which accounted for clustering effects at household level, only increasing age was an independent risk factor for CKD [OR=1.16 (1.07-1.25)]”, (Page 6)

**Reviewer comment: **Visual Aids: While the current figure is useful, additional visual aids, such as bar charts or scatter plots, could further clarify the distribution of CKD stages across communities.  

**Author response: ** A new figure showing the information requested has been included. (Figure 3)

**Reviewer comment: **Contextualization: Briefly contextualize the CKD prevalence data with national or regional statistics to highlight the severity.    

**Author response: **Revised as follows:

 The prevalence of CKD reported across Nigeria and Sub-Saharan Africa varies greatly depending on the population studied, CKD definition and methodology; from as low as 2% to >20% (Abd ElHafeez et al., 2018; Chukwuonye et al., 2018). However, our findings demonstrate a higher CKD prevalence in the nearest/semiurban community A, compared with a pooled prevalence of 10% and 13.7% reported for Africa and Nigeria, respectively (Abd ElHafeez et al., 2018; Raji et al., 2024). (Page 5)

**Social Determinants of CKD Risk**

**Reviewer comment: **Condensation: Consider condensing the list of behavioral factors by grouping similar items, such as unhealthy dietary habits.    

**Author response: **Revised as follows: 

 ‘Behavioural factors included unhealthy dietary habits (70-90%), low physical activity (47.2%), habitual exposure to potential nephrotoxins (37-44%)’. (Page 6)

**Reviewer comment: **Clearer Connections: Draw clearer connections between these social determinants and the higher CKD prevalence, emphasizing the compounding effect of multiple risk factors.    

**Author response: **Revised as follows:

 “The concentration of multiple social and environmental risk factors in the studied population may explain the high prevalence of CKD and other NCDs. These findings support the multicasuation theory, drawing attention to the need for a multi-faceted approach to CKD prevention.” (Page 6)

**Reviewer comment: **Implications: Discuss the potential public health implications of these social determinants in more depth.

**Author response: **Done as above. These points were further discussed in the section on implications for policy. (Pages  6,7)

**Reviewer comment: **Low Air Pollution Health Risk Literacy in Warri        

 Direct Linkage: Explicitly link low literacy levels to poor health outcomes, particularly in the context of CKD and air pollution.

**Author response: **Revised as follows: 

 “This low literacy level suggests that the necessary preventive measures such as individual behavioral changes, are lacking and this may contribute to the high burden of air pollution associated NCDs in the community”. (Page 7)

**Reviewer comment: **Awareness Campaigns: Suggest potential public health campaigns or educational interventions to improve awareness based on the findings.     

**Author response: **These have been suggested.

 “The high prevalence of CKD risk factors and low awareness of CKD and NCD status among the study participants suggests low health literacy and poor health-seeking behavior which necessitates more persuasive and inclusive public health educational interventions.”  (Page  7)

**Implications for Policy**

**Reviewer comment: **Structured Recommendations: Present the policy implications in a more structured format, possibly using bullet points or numbered lists for clarity.    

**Author response: **This section has been reviewed accordingly. (Page 7,8)

**Reviewer comment: **Evidence Integration: Integrate specific study findings more directly into the policy recommendations to strengthen the argument for immediate action.      

 Prioritization: Consider categorizing the recommendations into short-term, medium-term, and long-term actions.  

**Author response: **We appreciate your comments. This section has been improved as is shown in Table 1. (Page  8-11)

**Reviewer comment: **Stakeholder Engagement: Emphasize the role of specific stakeholders (e.g., government, NGOs, the public) in implementing these recommendations.

 Feasibility: Briefly discuss the feasibility of these recommendations, particularly in the context of existing infrastructure and resources.        

**Author response: **These points have all been considered in the revised table of recommendations. (Page 8-11)

**Limitations of the Study**

**Reviewer comment: **Strengths Emphasis: Counterbalance the limitations with a brief discussion of the study’s strengths, particularly its novel approach and the involvement of citizen scientists.  ** **  

**Author response: **This has been included as follows: 

 “Despite the limitations, the strength of the underlying study lies in the relatively large sample size, the extensive consideration and adjustment for confounding factors, the innovative combination of multiple research methods and involving citizen scientists in addressing a public health problem”. (Page 11)

**Reviewer comment: **Future Directions: Suggest specific areas for future research that could address these limitations, enhancing the study's impact.

**Author response: **This has been included as follows: 

 “More research is required from under-served areas to explore: this exposure-effect relationship, the mechanism of air pollution associated CKD and potential interventions to reverse it; and to develop educational interventions that would effectively improve public awareness of environmental health risks”. (Page 110)

**Conclusion**

**Reviewer comment: **Stronger Call to Action: End with a more forceful call to action directed at policymakers, highlighting the urgency of addressing CKD in the Niger Delta.    

**Author response: **Thank you for your suggestions. The section has been improved. (Page  11,12)

**Reviewer comment: **Summary of Key Points: Briefly summarize the most critical findings and recommendations to leave a lasting impression.    

**Author response: **Thank you for your suggestions. The section has been improved. (Page 11, 12)

**Overall**

**Reviewer comment: **Consistency: Ensure consistency in the use of terms (e.g., CKD vs. chronic kidney disease) throughout the document.    

**Author response: **CKD is now used consistently throughout the text, except for where it was first mentioned.   

**Reviewer comment: **Clarity: While the document is generally clear, some sections, particularly those with dense data, could benefit from simpler language and clearer transitions.    

**Author response: **Thank you. The authors have made significant efforts to improve the clarity and readability of the document using a proof reader.     

**Reviewer comment: **Visual Aids: Consider the strategic use of additional visual aids to break up text-heavy sections and improve reader engagement.    

**Author response: **Additional visual aids have been introduced. e.g., more figures and the recommendations have been tabulated.  

# Reviewer report: Reviewer response for version 1

**DOI:** 10.5256/f1000research.159925.r268338

**Published:** 2024-05-08

**Authors:** Chao Yang

**Affiliations:** 1 Peking University, Beijing, Beijing, China

This study provides insights into the association between air pollution and CKD risk in Niger Delta communities. Comprehensive policy interventions and public education are needed to mitigate the CKD risk and improve overall health outcomes in these communities. Here are several specific comments for the paper:

 1) The authors should provide a more detailed description of the mixed-methods approach used in the study. Clarify the specific quantitative and qualitative methods employed, sample sizes, and data collection techniques.

 2) The paper mentions that air pollutant concentrations were measured and compared to WHO limits. However, the analysis of these data and their statistical significance is lacking. The authors should include a more thorough analysis of the air pollution data.

 3) The paper discusses the association between ambient air pollution and CKD risk, but does not claim causality. The authors should clarify this distinction and discuss potential confounders or alternative explanations for the observed association.

 4) The paper should acknowledge other limitations of the study, such as sample size, data availability, or methodological constraints. Additionally, the authors should discuss potential directions for future research to build upon the current findings.

 5) The authors should discuss the generalizability of their findings to other communities and regions with similar industrial activities.

Does the paper provide a comprehensive overview of the policy and the context of its implementation in a way which is accessible to a general reader?

Partly

Is the discussion on the implications clearly and accurately presented and does it cite the current literature?

Partly

Are the recommendations made clear, balanced, and justified on the basis of the presented arguments?

Partly

Reviewer Expertise:

Public health

I confirm that I have read this submission and believe that I have an appropriate level of expertise to confirm that it is of an acceptable scientific standard, however I have significant reservations, as outlined above.

# Response

**Published:** 2024-06-17

**Authors:** Ogochukwu Okoye

**Affiliations:** Medicine, Delta State University, Abraka, Delta, Nigeria

Thank you for your kind critique of the manuscript. 

 All your comments are valid and appreciated. Being a policy brief, the manuscript was intentionally shortened to provide the basic information in a simple way for the intended audience, which includes policy makers. 

 More detailed Original manuscripts for a scientific audience  have been written and are currently undergoing peer review. However, the link to the Study Thesis was provided with this submission. 

 Thank you.

# Response

**Published:** 2024-09-30

**Authors:** Ogochukwu Okoye

**Affiliations:** Medicine, Delta State University, Abraka, Delta, Nigeria

**REVIEWER 1**

 We are grateful for your detailed and constructive critique which has helped to significantly improve the merit and quality of our manuscript

 1) **The authors should provide a more detailed description of the mixed-methods approach used in the study. Clarify the specific quantitative and qualitative methods employed, sample sizes, and data collection techniques.**

 Thank you for your observations.  Extensive methodological and statistical details were deliberately omitted as the manuscript was targeted at policymakers. However, we have provided additional details in the text.

 The underlying study was **an exploratory mixed methods study with an embedded citizen science inquiry**. Details of the first strand which was a focus group has been published and we have provided a reference to the article. In the current brief, we focus on findings from the other strands of the study.  

 (Pages 2,3,5)

 2) **The paper mentions that air pollutant concentrations were measured and compared to WHO limits. However, the analysis of these data and their statistical significance is lacking. The authors should include a more thorough analysis of the air pollution data.**

 For the reasons mentioned above, we omitted these details. However, some additional data have been provided in the text and as supplementary files.

 (Page 5, suppl 1, Figure 1)

 3) **The paper discusses the association between ambient air pollution and CKD risk, but does not claim causality. The authors should clarify this distinction and discuss potential confounders or alternative explanations for the observed association.**

 We agree with your observations. We have provided details of confounding factors considered and a supplementary file containing updated results from a binary mixed effects logistic model conducted to account for clustering effects.

 We report that long-term exposure to ambient air pollution is associated with CKD risk, but does not independently increase its risk, after adjusting for clustering effects and confounders (age, gender, hypertension, level of education and diabetes).

 (Page 6, suppl 2)

 4) **The paper should acknowledge other limitations of the study, such as sample size, data availability, or methodological constraints. Additionally, the authors should discuss potential directions for future research to build upon the current findings.**

 The section on limitation of the study has been improved and **suggestions for future research as follows**:

 “More research is required from under-served areas to explore: this exposure-effect relationship, the mechanism of air pollution associated CKD and potential interventions to reverse it; and to develop educational interventions that would effectively improve public awareness of environmental health risks”. 

 (Page 11)

 5) **The authors should discuss the generalizability of their findings to other communities and regions with similar industrial activities.**

 This has been included.

 “…. our findings are generalisable to similar vulnerable populations across the globe who reside near point emission sources” (Page 7)
