# Supplementary material for: Ambient Air Pollution and Chronic kidney disease risk in Deltan communities: A Policy Brief, 2023
Source: F1000Res. 2024 Nov 21;13:265. Originally published 2024 Apr 15. [Version 2] doi: 10.12688/f1000research.145904.2 (PMC11783039; doi:10.12688/f1000research.145904.2)
Supplement: Peer review history

# Response

**Published:** 2024-09-30

**Authors:** Ogochukwu Okoye

**Affiliations:** Medicine, Delta State University, Abraka, Delta, Nigeria

We are grateful for your detailed and constructive critique which has helped to significantly improve the merit and quality of our manuscript. 

**Comment:** This study proposes to discover association between air pollution and CKD.  Unfortunately, an accurate measure of air pollution was not available. 

 Consequently, attempts to examine the relationship do not have data of sufficient quality to make the linkage.

**Response:** Extensive methodological and statistical details were deliberately omitted as this document was targeted at policymakers. However, we have provided additional information in the text  to describe how air pollution was measured and other details are available in the link to the underlying study.  Page 4

**Comment: **Similarly, an accurate measure of the health outcome, kidney function was not available.

 Diagnosis of CKD requires two measures of kidney function (usually by eGRF) at least 3 months apart.

**Response:** We agree with your observation. The authors initially set out to obtain at least two measurements from participants who have abnormalities but encountered challenges with participant retention. This limitation has been highlighted in more detail. 

 However, all biological samples were handled and processed by a reputable independent laboratory with documented standard operating procedures to ensure accuracy and reliability.  Page 11

**Comment: **The paper was not conducted as a policy brief.  Large amounts of extant literature were missing from the evidence base presented **.**

**Response: **Thank you for your valid observation. We structured the paper according to the journal's template for a policy brief. Our intention was to provide a brief summary of the underlying study before delving into the policy implications and recommendations. We deliberately excluded excessive statistical information to enhance readability for the non-academic audience. However, we have now included more data in the text figures and supplementary files. 

**Comment: **The paper has a limited data reference list.

**Response: **The short reference list was to adhere to the limits of a policy brief. More references have been included in specific sections. Pages 3,5,6,7,14

**Comment: **Abstract mentions CKDu in passing without mentioning its prevalence in certain LMIC settings.  The disease CKDu in Sri Lankan and Mesoamerican settings.

**Response: **Thank you for your comment. There may have been a mix up. CKDu was not mentioned in the abstract.

 -

# Response

**Published:** 2024-09-30

**Authors:** Ogochukwu Okoye

**Affiliations:** Medicine, Delta State University, Abraka, Delta, Nigeria

We are grateful for your detailed and constructive critique which has helped to improve the merit and quality of our manuscript.

 ABSTRACT

**Reviewer comment: **Objective Clarification: Include a brief statement that clearly outlines the study's objective.

**Author response: **The objective has been clarified as follows:

 “This brief is based on a mixed-methods study conducted in four communities situated near an oil and gas refinery in Warri, Nigeria to explore perceived and actual air pollution risk and determine whether long-term exposure to AAP increases NCD risk” (Page 2)

**Reviewer comment: **Methodology Mention: Explicitly mention that a mixed-methods approach was used.

**Author response: **Done as stated above

**Reviewer comment: **Specific Findings: Highlight specific data points, such as the exact levels of pollutants that exceed WHO limits.

**Author response: **Included as follows:

 “Measured PM _2.5_ ranged from 22.8 – 28.0 μg/m3, PM _10_, 40.6 - 55.5 μg/m3, and CO _2_, 584-652ppm”. 2

**Reviewer comment: **Policy Recommendations: Summarise one or two key policy recommendations for a clear takeaway.

**Author response: **Included as follows: 

 “The government needs to urgently invest in air monitoring services and data, cleaner technologies and environmental risk communication through various media channels. We strongly recommend public inclusion in planning, designing and implementing future environmental interventions. Lastly, environmental risk factors such as air pollution should feature prominently in strategic plans for NCD prevention”. (Page 2)

**Introduction**

**Reviewer comment: **Streamlining: Consider consolidating the discussion on traditional CKD risk factors to avoid redundancy.

 Focused Transition: Ensure a smooth transition from discussing CKD globally to the specific context of the Niger Delta.

 Identification of Gap: Introduce the research gap on the link between air pollution and CKD in the Niger Delta earlier in the section.

**Author response: **Thank you for your suggestions. We have reviewed this section accordingly. (Page 3)

**Reviewer comment: **Policy Outcomes and Implications

**Author response: **This section has been improved accordingly

**Reviewer comment: **Direct Link: Explicitly connect the absence of CKD in national health policy to the study’s findings on air pollution and CKD prevalence.

**Author response: **This has been revised as follows: “This lack of recognition of CKD and the environmental risk factors important in the disease epidemiology, may explain why CKD and related NCDs are persistent.” (Page 4)

**Reviewer comment: **Broader Implications: Discuss the broader public health implications if this policy gap continues, including potential long-term consequences.

**Author response: **“The long-term consequences of continually neglecting CKD in health policies include the persistence of hypertension and CKD with associated considerable morbidity and mortality; high health care expenditure which further impoverishes the affected members of society and their families”. (Page 4)

**Reviewer comment: **Evidence of High Ambient Air Pollutant Levels in Warr

 Narrative Flow: Integrate the table with the narrative by referencing key figures directly in the text.

**Author response: **This section has been improved accordingly and highlighted in the text (Page 4-5)

**Reviewer comment: **Benchmark Against Global Standards: Briefly compare the findings with global or regional averages for context.

**Author response: **This section has been improved as follows: 

 “The PM2.5 concentrations for three of the communities are higher than Nigeria’s 2023 average of 23.9 μg/m3 (IQAIR) and five times higher than the WHO acceptable limit. Furthermore, these levels are much higher than 7.8μg/m3 achieved in Angola, the least polluted African country which ranked 114th out of 134 countries, while Nigeria was ranked the 35th most polluted”.( Page 4-5)

**Reviewer comment: **Graphical Representation: Consider using a graph to visualize the levels of pollutants across communities for greater impact.

**Author response: **A bar chart showing air pollutant levels in the 4 communities have been included. (Figure 1)

**Reviewer comment: **High Prevalence of CKD and the Risk Factors

 Risk Factor Hierarchy: Present the risk factors in order of their strength of association with CKD.    

**Author response: **Included as follows: 

 “The independent risk factors for CKD were proximity to the refinery [OR=2.00 (1.43 – 2.81], increasing age [OR=1.02(1.005-1.04)], hypertension [OR=1.61(1.12-2.31)], and level of education [OR=0.63(0.44-0.91)]. In a further logistic mixed model, which accounted for clustering effects at household level, only increasing age was an independent risk factor for CKD [OR=1.16 (1.07-1.25)]”, (Page 6)

**Reviewer comment: **Visual Aids: While the current figure is useful, additional visual aids, such as bar charts or scatter plots, could further clarify the distribution of CKD stages across communities.

**Author response: **A new figure showing the information requested has been included. (Figure 3)

**Reviewer comment: **Contextualization: Briefly contextualize the CKD prevalence data with national or regional statistics to highlight the severity.

**Author response: **Revised as follows:

 The prevalence of CKD reported across Nigeria and Sub-Saharan Africa varies greatly depending on the population studied, CKD definition and methodology; from as low as 2% to >20% (Abd ElHafeez et al., 2018; Chukwuonye et al., 2018). However, our findings demonstrate a higher CKD prevalence in the nearest/semiurban community A, compared with a pooled prevalence of 10% and 13.7% reported for Africa and Nigeria, respectively (Abd ElHafeez et al., 2018; Raji et al., 2024). (Page 5)

**Social Determinants of CKD Risk**

**Reviewer comment: **Condensation: Consider condensing the list of behavioral factors by grouping similar items, such as unhealthy dietary habits.

**Author response: **Revised as follows: 

 ‘Behavioural factors included unhealthy dietary habits (70-90%), low physical activity (47.2%), habitual exposure to potential nephrotoxins (37-44%)’. (Page 6)

**Reviewer comment: **Clearer Connections: Draw clearer connections between these social determinants and the higher CKD prevalence, emphasizing the compounding effect of multiple risk factors.

**Author response: **Revised as follows:

 “The concentration of multiple social and environmental risk factors in the studied population may explain the high prevalence of CKD and other NCDs. These findings support the multicasuation theory, drawing attention to the need for a multi-faceted approach to CKD prevention.” (Page 6)

**Reviewer comment: **Implications: Discuss the potential public health implications of these social determinants in more depth.

**Author response: **Done as above. These points were further discussed in the section on implications for policy. (Pages 6,7)

**Reviewer comment: **Low Air Pollution Health Risk Literacy in Warri

 Direct Linkage: Explicitly link low literacy levels to poor health outcomes, particularly in the context of CKD and air pollution.

**Author response: **Revised as follows: 

 “This low literacy level suggests that the necessary preventive measures such as individual behavioral changes, are lacking and this may contribute to the high burden of air pollution associated NCDs in the community”. (Page 7)

**Reviewer comment: **Awareness Campaigns: Suggest potential public health campaigns or educational interventions to improve awareness based on the findings.

**Author response: **These have been suggested.

 “The high prevalence of CKD risk factors and low awareness of CKD and NCD status among the study participants suggests low health literacy and poor health-seeking behavior which necessitates more persuasive and inclusive public health educational interventions.” (Page 7)

**Implications for Policy**

**Reviewer comment: **Structured Recommendations: Present the policy implications in a more structured format, possibly using bullet points or numbered lists for clarity.

**Author response: **This section has been reviewed accordingly. (Page 7,8)

**Reviewer comment: **Evidence Integration: Integrate specific study findings more directly into the policy recommendations to strengthen the argument for immediate action.

 Prioritization: Consider categorizing the recommendations into short-term, medium-term, and long-term actions.

**Author response: **We appreciate your comments. This section has been improved as is shown in Table 1. (Page 8-11)

**Reviewer comment: **Stakeholder Engagement: Emphasize the role of specific stakeholders (e.g., government, NGOs, the public) in implementing these recommendations.

 Feasibility: Briefly discuss the feasibility of these recommendations, particularly in the context of existing infrastructure and resources.

**Author response: **These points have all been considered in the revised table of recommendations. (Page 8-11)

**Limitations of the Study**

**Reviewer comment: **Strengths Emphasis: Counterbalance the limitations with a brief discussion of the study’s strengths, particularly its novel approach and the involvement of citizen scientists.

**Author response: **This has been included as follows: 

 “Despite the limitations, the strength of the underlying study lies in the relatively large sample size, the extensive consideration and adjustment for confounding factors, the innovative combination of multiple research methods and involving citizen scientists in addressing a public health problem”. (Page 11)

**Reviewer comment: **Future Directions: Suggest specific areas for future research that could address these limitations, enhancing the study's impact.

**Author response: **This has been included as follows: 

 “More research is required from under-served areas to explore: this exposure-effect relationship, the mechanism of air pollution associated CKD and potential interventions to reverse it; and to develop educational interventions that would effectively improve public awareness of environmental health risks”. (Page 110)

**Conclusion**

**Reviewer comment: **Stronger Call to Action: End with a more forceful call to action directed at policymakers, highlighting the urgency of addressing CKD in the Niger Delta.

**Author response: **Thank you for your suggestions. The section has been improved. (Page 11,12)

**Reviewer comment: **Summary of Key Points: Briefly summarize the most critical findings and recommendations to leave a lasting impression.

**Author response: **Thank you for your suggestions. The section has been improved. (Page 11, 12)

**Overall**

**Reviewer comment: **Consistency: Ensure consistency in the use of terms (e.g., CKD vs. chronic kidney disease) throughout the document.

**Author response: **CKD is now used consistently throughout the text, except for where it was first mentioned.

**Reviewer comment: **Clarity: While the document is generally clear, some sections, particularly those with dense data, could benefit from simpler language and clearer transitions.

**Author response: **Thank you. The authors have made significant efforts to improve the clarity and readability of the document using a proof reader.

**Reviewer comment: **Visual Aids: Consider the strategic use of additional visual aids to break up text-heavy sections and improve reader engagement.

**Author response: **Additional visual aids have been introduced. e.g., more figures and the recommendations have been tabulated.

# Response

**Published:** 2024-09-30

**Authors:** Ogochukwu Okoye

**Affiliations:** Medicine, Delta State University, Abraka, Delta, Nigeria

**REVIEWER 1**

 We are grateful for your detailed and constructive critique which has helped to significantly improve the merit and quality of our manuscript

 1) **The authors should provide a more detailed description of the mixed-methods approach used in the study. Clarify the specific quantitative and qualitative methods employed, sample sizes, and data collection techniques.**

 Thank you for your observations.  Extensive methodological and statistical details were deliberately omitted as the manuscript was targeted at policymakers. However, we have provided additional details in the text.

 The underlying study was **an exploratory mixed methods study with an embedded citizen science inquiry**. Details of the first strand which was a focus group has been published and we have provided a reference to the article. In the current brief, we focus on findings from the other strands of the study.  

 (Pages 2,3,5)

 2) **The paper mentions that air pollutant concentrations were measured and compared to WHO limits. However, the analysis of these data and their statistical significance is lacking. The authors should include a more thorough analysis of the air pollution data.**

 For the reasons mentioned above, we omitted these details. However, some additional data have been provided in the text and as supplementary files.

 (Page 5, suppl 1, Figure 1)

 3) **The paper discusses the association between ambient air pollution and CKD risk, but does not claim causality. The authors should clarify this distinction and discuss potential confounders or alternative explanations for the observed association.**

 We agree with your observations. We have provided details of confounding factors considered and a supplementary file containing updated results from a binary mixed effects logistic model conducted to account for clustering effects.

 We report that long-term exposure to ambient air pollution is associated with CKD risk, but does not independently increase its risk, after adjusting for clustering effects and confounders (age, gender, hypertension, level of education and diabetes).

 (Page 6, suppl 2)

 4) **The paper should acknowledge other limitations of the study, such as sample size, data availability, or methodological constraints. Additionally, the authors should discuss potential directions for future research to build upon the current findings.**

 The section on limitation of the study has been improved and **suggestions for future research as follows**:

 “More research is required from under-served areas to explore:  this exposure-effect relationship, the mechanism of air pollution associated CKD and potential interventions to reverse it; and to develop educational interventions that would effectively improve public awareness of environmental health risks”. 

 (Page 11)

 5) **The authors should discuss the generalizability of their findings to other communities and regions with similar industrial activities.**

 This has been included.

 “…. our findings are generalisable to similar vulnerable populations across the globe who reside near point emission sources” (Page 7)
